# Evaluation of the SMS-ICU score for multicontinental use: A federated external validation study using critical care registry data

**DOI:** 10.1016/j.ccrj.2026.100206

**Published:** 2026-08-13

**Authors:** Alexander Tracy, Fathima Fazla, Jorge I.F Salluh, Rashan Haniffa, Rabiul Alam Md Erfan Uddin, Diptesh Aryal, Sylvia Brinkman, Gastón Burghi, Zakary Doherty, Dave Dongelmans, Stefano Finazzi, Ville Ihalainen, Bharath Kumar Tirupakuzhi Vijayaraghavan, Mohammed Basri Mat-Nor, Tahlia Perumal, David Pilcher, Luigi Pisani, Matti Reinikainen, Moses Siaw-Frimpong, Menbeu Sultan, David Thomson, Giovanni Tricella, Abigail Beane, Otavio Ranzani

**Affiliations:** a Usher Institute, University of Edinburgh, Edinburgh, United Kingdom; b National Intensive Care Surveillance-MORU, Colombo, Sri Lanka; c Instituto D’Or de Pesquisa e Ensino Rio de Janeiro, Brazil; d Postgraduate Program, Instituto D’Or de Pesquisa e Ensino Rio de Janeiro, Brazil; e Centre for Inflammation Research, University of Edinburgh, Edinburgh, United Kingdom; f Department of Medicine, Chittagong Medical College, Chittagong, Bangladesh; g Nepal Intensive Care Research Foundation (NICRF), Nepal; h National Intensive Care Evaluation (NICE) Foundation, Amsterdam, the Netherlands; i Department of Medical Informatics, Amsterdam UMC, University of Amsterdam, and Amsterdam Public Health, Methodology and Digital Health, Amsterdam, the Netherlands; j Intensive Care Unit, Hospital Maciel, Montevideo, Montevideo, Uruguay; k Department of Intensive Care Medicine, Amsterdam UMC, University of Amsterdam, Amsterdam, the Netherlands; l Mario Negri Institute for Pharmacological Research IRCCS, Laboratory of Clinical Data Science, Department of Medical Epidemiology, Lombardia, Italy; m Department of Anaesthesiology and Intensive Care, Kuopio University Hospital, and University of Eastern Finland, Kuopio, Finland; n Department of Critical Care Medicine, Apollo Hospitals, Chennai, India; o Department of Anaesthesiology and Intensive Care, International Islamic University Malaysia, Kuala Lumpur, Malaysia; p Division of Pulmonology, Department of Medicine, Centre for Lung Infection and Immunity, University of Cape Town Lung Institute, Cape Town, South Africa; q Centre for the Study of Antimicrobial Resistance, South African Medical Research Council and University of Cape Town, Cape Town, South Africa; r The Australian and New Zealand Intensive Care (ANZICS) Centre for Outcome and Resource Evaluation, Melbourne, Australia; s Australian and New Zealand Intensive Care Research Centre, Monash University, Melbourne, Australia; t The Alfred Intensive Care Academic Centre, Bayside Health, Melbourne, Australia; u Department of Precision-Regenerative Medicine and Jonic Area (DiMePRe-J), Section of Anesthesiology and Intensive Care Medicine, University of Bari "Aldo Moro", Bari, Italy; v Mahidol Oxford Tropical Research Unit (MORU), Bangkok, Thailand; w Department of Anaesthesia and Intensive Care, Komfo Anokye Teaching Hospital, Kumasi, Ghana; x Department of Emergency Medicine and Critical Care, St. Paul's Hospital Millennium Medical College, Addis Ababa, Ethiopia; y Department of Anaesthesia and Peri-operative Medicine, Division of Critical Care, University of Cape Town, Groote Schuur Hospital, Cape Town, South Africa; z DataHealth Lab, Institut de Recerca Sant Pau (IR SANT PAU), Barcelona, Spain; aa Pulmonary Division, Faculty of Medicine, Heart Institute, Hospital das Clinicas da Faculdade de Medicina da Universidade de Sao Paulo, Sao Paulo, Brazil

**Keywords:** Mortality, Intensive care, Registries, Global health, Prognosis, Severity of illness, Severity of illness index

## Abstract

**Objective:**

To evaluate the suitability of the Simplified Mortality Score for the Intensive Care Unit (SMS-ICU) for use in critical care registries across multiple continents.

**Design:**

A federated external validation study.

**Setting:**

Intensive care units (ICUs) belonging to 12 critical care registries on five continents.

**Participants:**

Individuals over the age of 18 years who were admitted to participating ICUs during the 2023 calendar year. Readmissions within the same hospital encounter and interhospital transfers were excluded.

**Interventions:**

None.

**Main outcome measures:**

The primary outcome was all-cause in-hospital mortality. A secondary analysis considered ICU mortality.

**Results:**

12 registries participated (three from Africa, four from Asia, one from Oceania, two from Europe and two from South America). In total, 494,705 admissions were included from 546 ICUs in 13 countries. Seven registries systematically reported hospital outcomes and were included in the primary analysis. Discrimination was acceptable (pooled area under the receiver operating characteristic curve [AUROC] 0.76 [95% CI 0.72–0.80]). Calibration and overall model fit varied between registries (Brier scores 0.09–0.14). In the secondary analysis of ICU outcomes in all 12 registries, discrimination remained acceptable but with higher variation between registries (pooled AUROC 0.73 [0.69–0.77]). Calibration and overall model fit also varied widely (Brier scores 0.07–0.46).

**Conclusions:**

SMS-ICU may be used as an index of illness severity in international intensive care populations. This may facilitate international collaborative research and synthesis of findings in systematic reviews and meta-analyses.

## 1 Introduction

Illness severity scores and prognostic models serve multiple purposes in critical care.^1^ These include describing baseline illness severity in research participants, benchmarking intensive care unit (ICU) performance and evaluating quality improvement initiatives.^2^

Many challenges faced by ICUs have international scope, including pandemics, natural disasters and sociodemographic change. As a result, there is increasing interest in strengthening international collaboration in the field.[3], [4], [5] These efforts would be aided by the ability to apply scoring systems internationally. For example, in collaborative international research, this would allow for harmonised description of illness severity across registry datasets and trial populations. It would also enable the synthesis of international illness severity-stratified results by systematic reviews and meta-analyses.^5^

Unfortunately, many widely used models vary in their applicability to the full range of geographical and socio-economic contexts.^2^^,^^6^ The use of many such scores entails a significant data collection burden, and their performance is particularly inconsistent in lower-income settings.^7^^,^^8^ There are multiple explanations for this, including limited data availability for certain variables and outcomes.^7^^,^^9^ Because the mechanisms underlying data missingness differ between settings, this problem cannot easily be solved by a universal imputation strategy.^6^

One potential solution to this problem is the use of ‘simplified’ scoring systems restricted to variables for which data are widely available. Examples include the severity assessment score (SEVERITAS), Rwanda Mortality Probability Model (R-MPM), Tropical Intensive Care (TropICS) score and the Simplified Mortality Score for the ICU (SMS-ICU).^8^^,^[10], [11], [12]

Users of ‘simplified’ scores accept reduced predictive performance in exchange for improved utility across diverse settings. Consequently, when applied within individual countries, predictive performance will be inferior to that of locally calibrated models, *e.g.* the Australian and New Zealand Risk of Death (ANZROD) model.^13^ Therefore, they should not replace locally calibrated models for the evaluation of individual ICU performance.

SMS-ICU is calculated in the first 24 h of ICU admission using seven easily available variables. It was originally derived in clinical trial populations to predict 90-day mortality among acutely ill adults.^11^ The score has since been customised to predict in-hospital mortality in a large Brazilian cohort.^14^ Notably, it has not been validated for use in elective ICU admissions. This study aims to evaluate the suitability of this customised SMS-ICU score for use in critical care registries across multiple continents.

## 2 Methods

### 2.1 Study design, setting and participants

This retrospective federated external validation study was conducted using contemporaneous data from critical care registries on multiple continents.^15^ Registries were identified from membership of international collaborations, including Linking of Global Intensive Care (LOGIC) and Collaboration for Research Implementation and Training for Critical Care in Asia and Africa (CCAA).^3^^,^^16^^,^^17^ Additional registries known to LOGIC or the authors were invited to participate.

This study included patients admitted to ICUs during 2023. In Finland, patients admitted during 2022 were included instead due to internal data access restrictions. ICUs were eligible for inclusion if they started contributing data to their registry at least one year prior to the start of the study period. This provided a lag period of at least one year to maximise data quality.

As per the studies that developed and customised SMS-ICU, patients were excluded if they were: not over 18 years of age at ICU admission, without a known survival status for ICU or hospital admission, or transferred to another hospital.^11^^,^^14^ A sensitivity analysis excluded all interhospital transfers, both inward and outward. This accounted for the possibility that inward inter-ICU transfers may occur beyond the initial 24-h period during which SMS-ICU should be calculated.^11^ Unlike previous studies, elective ICU admissions were not excluded.

Central ethical approval was provided by the Alfred Hospital Ethics Committee (project no. 514/24) on 16th August 2024. Necessary local administrative approvals were obtained by each participating registry.

This study is reported according to TRIPOD guidelines.^18^

### 2.2 Federated approach

Analysis materials were prepared and distributed to participating registries. These included instructions for variable mapping, data dictionary, analysis scripts and study protocol (all available from https://github.com/NICST-PROTECT/sms-icu-validation/tree/main). The analysis scripts automatically applied study inclusion and exclusion criteria. Variable mapping, data processing and analysis were conducted internally by each registry, and aggregate results were reported without transfer of patient-level data.

### 2.3 Variables

The SMS-ICU component variables are age, blood pressure, admission type, haematological malignancy or metastatic cancer, vasopressor or inotrope use, respiratory support, and renal replacement therapy (Supplementary Table 1). SMS-ICU variables were defined according to the original study, as outlined in the data dictionary supplied to participating registries.^11^ The supplementary methods section describes any local adaptations to the original variable handling (Supplementary Methods).

To describe case-mix, registries were requested to report the reason for ICU admission using APACHE II diagnostic categories. However, alternative categorisations were permitted if harmonisation with APACHE II categories was not feasible. This maximised the range of registries able to participate.

The main outcome of interest was all-cause in-hospital mortality. Therefore, the primary analysis compared SMS-ICU predicted mortality to actual in-hospital mortality. Only registries that systematically collected hospital mortality outcomes were included in this analysis. In registries where only a subset of ICUs systematically reported hospital outcomes, the registries were filtered to include only ICUs with <20% hospital outcome missingness.

As all registries systematically collected ICU mortality outcomes, an *a priori* secondary analysis was performed comparing predicted mortality to actual ICU mortality. The purpose of this analysis was to test score performance in a broader range of settings, including those in which hospital outcomes are not yet systematically collected.

### 2.4 Sample size

Previous work has suggested that 100–200 outcome events are required to externally validate a predictive model.^19^^,^^20^ All eligible patients from each registry were included, and it was expected that this threshold would be met in all participating registries.

### 2.5 Statistical analysis

SMS-ICU scores (see Supplementary Table 1) were calculated.^11^ The customised equation for prediction of hospital mortality^14^ was used to predict outcomes for both primary and secondary analyses. All analyses were conducted in *R* version 4.4.

To assess discrimination, receiver operating characteristic (ROC) curves were plotted using the *pROC* package and the area under the curve (AUROC) was reported.^21^ The *a priori* threshold for acceptable discrimination was set at 0.7, in line with a commonly used definition.^22^ This threshold is expected to reflect adequate discrimination for international use; excellent discrimination is not required for this purpose. AUROCs from each registry were pooled using restricted maximum likelihood meta-analysis with the *metafor* package.^23^^,^^24^

Calibration curves were plotted with the *rms* package using both logistic and non-parametric LOWESS-smoothed calibration curves. Calibration-in-the-large was assessed using the calibration intercept. Both the calibration intercept and calibration slope were derived from the logistic calibration function. Observed/expected (O/E) ratios (equivalent to standardised mortality ratios) were reported for each registry.^25^

Overall model fit was assessed by calculation of Brier scores in each registry.^26^

Sensitivity analyses consisted of: (i) exclusion of both inward and outward interhospital transfers as described above, and (ii) a complete case analysis (see “Missing Data” below).

Subgroup analyses were performed for medical (non-surgical), emergency surgical, and elective surgical admissions. These subgroups were selected because SMS-ICU's applicability to elective cases was unknown. Adequate performance in each of these subgroups is a prerequisite for broad application across critical care populations.

A further subgroup analysis was conducted for patients receiving invasive mechanical ventilation. This aimed to assess score performance in a group with higher illness severity and risk of death.^27^

Forest plots comparing AUROC between subgroups were drawn using *ggplot2*.

### 2.6 Missing data

Missing data for SMS-ICU component variables were handled by modal imputation using *R*. Modal imputation was preferred to normal imputation because some non-scoring SMS-ICU categories, *e.g.* admission type, were expected to apply to a minority of patients. Multiple imputation was not used due to the expected low missingness rate, the low number of variables available for inclusion in a multiple imputation model, and the methodological complexity of implementing this in a federated study. A sensitivity analysis considered only complete cases with no imputation.

Missing outcomes were handled by excluding individuals for whom the outcome was missing.

## 3 Results

### 3.1 Study population

In total, 40 critical care registries were approached to participate. 21 registries expressed an initial interest in participation, of which 9 did not proceed. 12 registries representing 13 countries from five continents agreed to participate and completed the analysis. These are described in Supplementary Table 2. The original study populations for development, customisation and validation of the SMS-ICU score are presented in Supplementary Table 3 for comparison.^11^^,^^14^

### 3.2 In-hospital mortality analysis

#### 3.2.1 Primary in-hospital mortality analysis

The study population for the in-hospital mortality analysis is described in Table 1. In total, 447,350 admissions were included from 512 ICUs in nine countries. Hospital mortality ranged from 8.1% to 21.2%. The median missingness rate for SMS-ICU variables was 0.1% (IQR 0–0.2%) (Table 1). Case-mix for each registry is described in Supplementary Table 4a–g.

**Table 1:** Characteristics of the study population for the primary (in-hospital mortality) analysis.^a^

Registry location		Aus & NZ	Brazil	Finland	India	Nepal	Netherlands	Uruguay
Number of ICUs included		194	207	25	11	1	68	6
Total ICU admissions during study period		197 713	180 268	16 460	7610	822	70 080	2240
Number of admissions included		183 444	176 866	13 573	6615	621	64 163	2068
Number of exclusions	Total patients excluded	14 269	3402	2887	995	201	5917	172
	Age not over 18 years	1592	1300	998	322	24	414	22
	Readmission to ICU within same hospital encounter	8245	0	1472	213	40	3163	0
	Interhospital transfer	3685	2102	416	77	34	2340	150
	Missing outcome	747	0	1	383	103	0	0
Median patient age (IQR)		66 (52–76)	65 (47–78)	65 (53–73)	60 (48–70)	68 (51–79)	66 (55–74)	64 (49–74)
Median systolic blood pressure (IQR)		97 (88–108)	121 (105–140)	90 (82–102)	120 (110–140)	130 (112–151)	91 (80–105)	120 (100–142)
Number of male patients (% of total)		111 525 (56.4)	85 743 (48.5)	8578 (63.2)	4064 (61.4)	345 (55.6)	42 839 (61.1)	1225 (59.2)
Number of patients admitted after emergency surgery (% of total)		28 933 (15.8)	12 602 (7.1)	2446 (18.0)	87 (1.3)	15 (2.4)	7369 (11.5)	224 (10.8)
Number of patients admitted after elective surgery (% of total)		69 482 (37.9)	31 899 (18.0)	3552 (26.2)	1019 (15.4)	92 (14.8)	22 615 (35.2)	423 (20.4)
Number of patients receiving infused vasopressors/inotropes (% of total)		70 527 (38.4)	25 473 (14.4)	8232 (60.7)	1294 (19.6)	67 (10.8)	31 223 (48.7)	461 (22.3)
Number of patients receiving NIV/CPAP (% of total)		18 873 (10.3)	20 583 (11.6)	1050 (7.1)	564 (10.4)	30 (4.8)	NA^b^	NA
Number of patients receiving invasive mechanical ventilation (% of total)		55 687 (30.4)	12 602 (7.1)	6100 (44.9)	1457 (22.0)	57 (9.2)	NA	718 (34.7)
Number of patients receiving RRT (% of total)		7043 (3.8)	4889 (2.8)	774 (4.7)	283 (4.3)	7 (1.1)	2172 (3.4)	65 (3.1)
Total number of in-hospital deaths (% of total)		14 864 (8.1)	22 382 (12.7)	1636 (12.1)	1022 (15.4)	75 (12.1)	8384 (13.1)	439 (21.2)
Prevalence of missing data for study variables	Blood pressure (% of total)	0 (0)	2549 (1.4)	210 (1.6)	10 (0.15)	0 (0)	482 (0.8)	84 (4.1)
	Metastasis/haematological malignancy (% of total)	0 (0)	81 (0.05)	692 (5.9)	0 (0)	0 (0)	0 (0)	3 (0.1)
	Vasopressor/inotrope use (% of total)	11 747 (6.4)	204 (0.1)	0 (0)	10 (0.2)	0 (0)	0 (0)	2 (0.1)
	Respiratory support (% of total)	0 (0)	204 (0.1)	1544 (11.4)	10 (0.2)	0 (0)	0 (0)	2 (0.1)
	Renal replacement therapy (% of total)	13 037 (7.1)	204 (0.1)	0 (0)	10 (0.2)	0 (0)	36 195 (56.4)^c^	2 (0.1)
	Admission type (% of total)	0 (0)	0 (0)	29 (0.2)	0 (0)	0 (0)	201 (0.3)	0 (0)

a Participating registries were excluded from this analysis if they did not systematically report hospital mortality. For the registries in India and Nepal, individual ICUs were excluded from this analysis if they did not systematically report hospital mortality.

b ‘NA’ refers to data that are not collected by the relevant registry. The Netherlands registry does not report mode of ventilatory support (*i.e*. invasive versus non-invasive).

c Renal replacement therapy (RRT) data are only reported by a subset of ICUs in the Netherlands. In the analysis, RRT data were used where available and modal imputation was applied where not available.

**Fig. 1:**
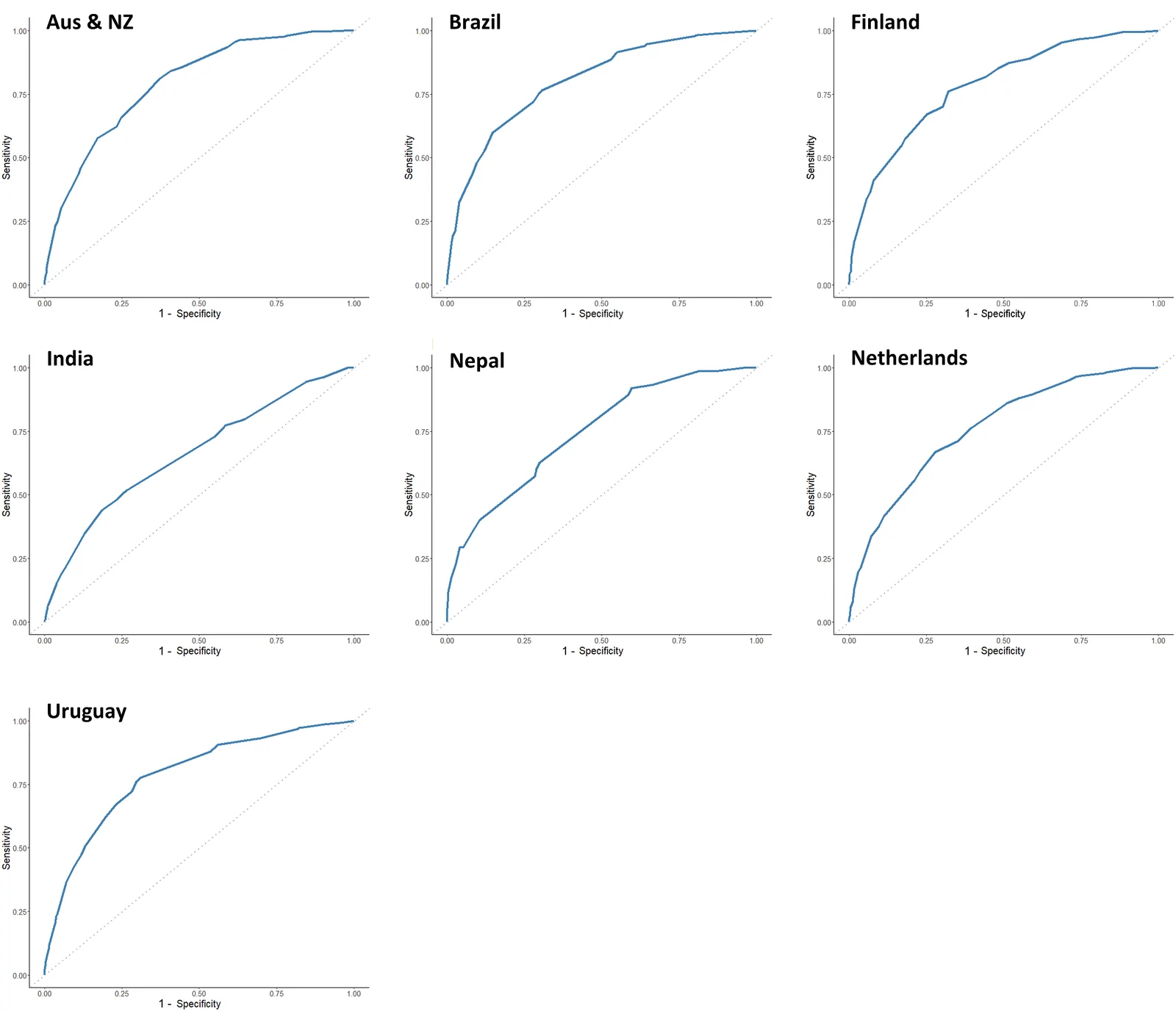
**ROC curves (solid blue) presented by registry for the primary (in-hospital mortality) analysis**. Sensitivity is plotted on the y axis, against (1 – specificity) on the x axis.

Discrimination of SMS-ICU, assessed using ROC curves, is shown for each registry in Fig. 1. AUROC met the pre-specified threshold of 0.7 in six of seven registries (Table 2). The pooled AUROC was 0.76 (95% CI 0.72–0.80).

**Table 2:** Results from the primary (in-hospital mortality) analysis.

	Aus & NZ	Brazil	Finland	India	Nepal	Netherlands	Uruguay
AUROC (95% CI)^a^	0.79 (0.79–0.8)	0.8 (0.8–0.81)	0.78 (0.77–0.79)	0.66 (0.64–0.68)	0.75 (0.69–0.8)	0.76 (0.76–0.77)	0.79 (0.76–0.81)
Calibration slope^b^	0.9225	1.0095	0.9397	0.5536	1.045	0.8159	0.8445
Calibration intercept^c^	−1.2509	−0.2486	−1.0363	−0.5829	−0.0511	−1.1667	0.0026
O/E ratio (95% CI)^d^	0.42 (0.41–0.43)	0.83 (0.82–0.84)	0.5 (0.47–0.52)	1.13 (1.07–1.18)	0.91 (0.74–1.12)	0.49 (0.48–0.51)	1.12 (1.05–1.21)
Brier score^e^	0.0873	0.0916	0.113	0.125	0.0921	0.1282	0.136

a AUROC is the area under the receiver operating characteristic.

b Calibration slope and intercept refer to properties of the logistic calibration function. The calibration slope indicates the degree of proportional agreement between predicted and observed outcomes; an ideal calibration curve has a calibration slope of 1.

c The calibration intercept quantifies systematic prediction bias, giving an assessment of calibration-in-the-large. An ideal calibration curve has an intercept of 0.

d The O/E ratio is the ratio of observed to expected events, equivalent to the standardised mortality ratio.

e Brier score ranges from 0 to 1, with values closer to 0 representing better model fit.

**Fig. 2:**
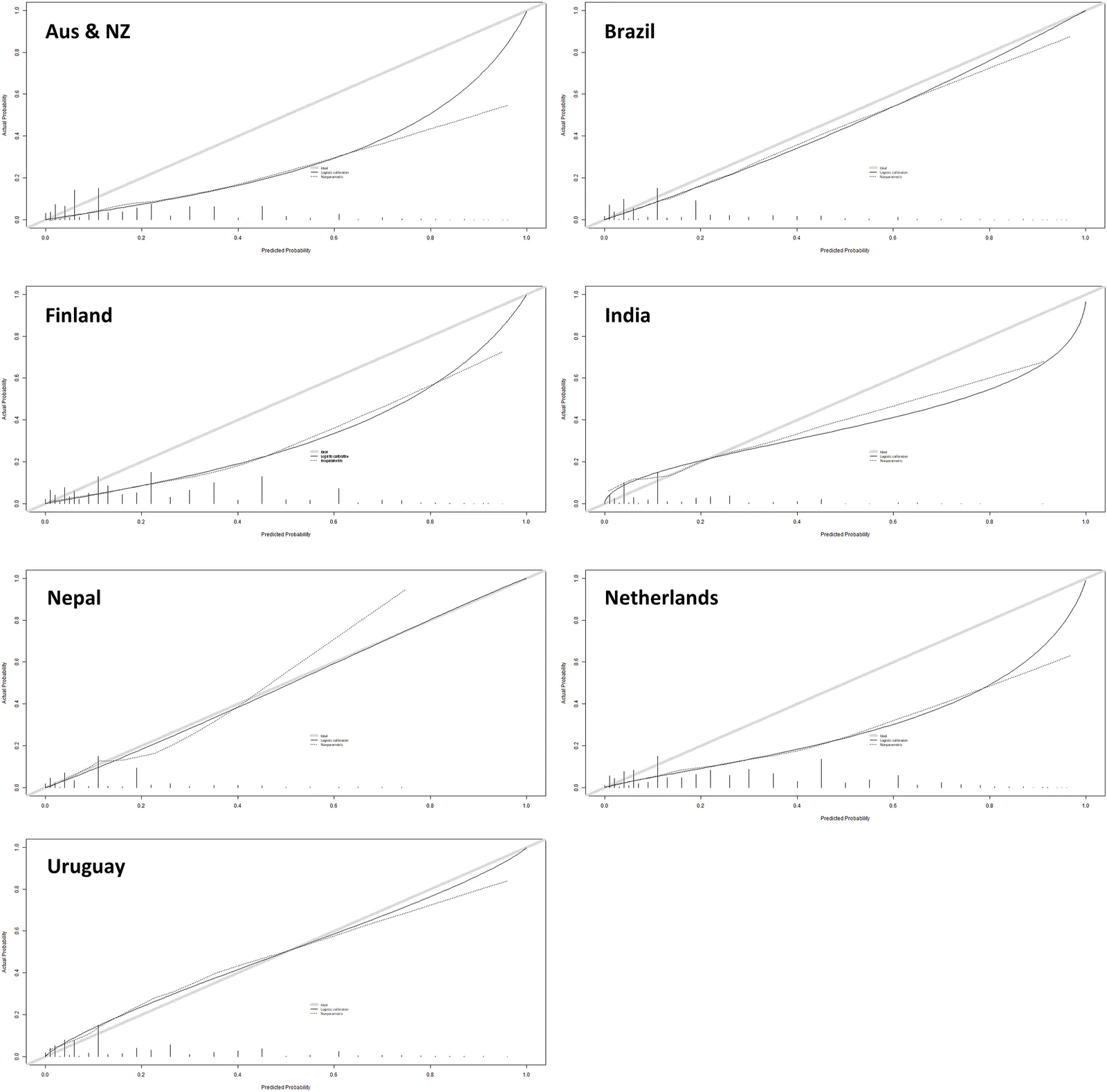
**Calibration curves for primary (in-hospital mortality) analysis**. Observed probability (y-axis) is plotted against predicted probability (x-axis). Logistic calibration curves (solid black) and nonparametric smoothed (dotted) calibration curves are plotted.

Calibration varied between registries (Fig. 2, Table 2), with O/E ratios ranging from 0.42 to 1.13 (Table 2).

Overall model fit is represented by Brier scores ranging from 0.087 to 0.136 (Table 2).

#### 3.2.2 Sub-group in-hospital mortality analysis

**Fig. 3:**
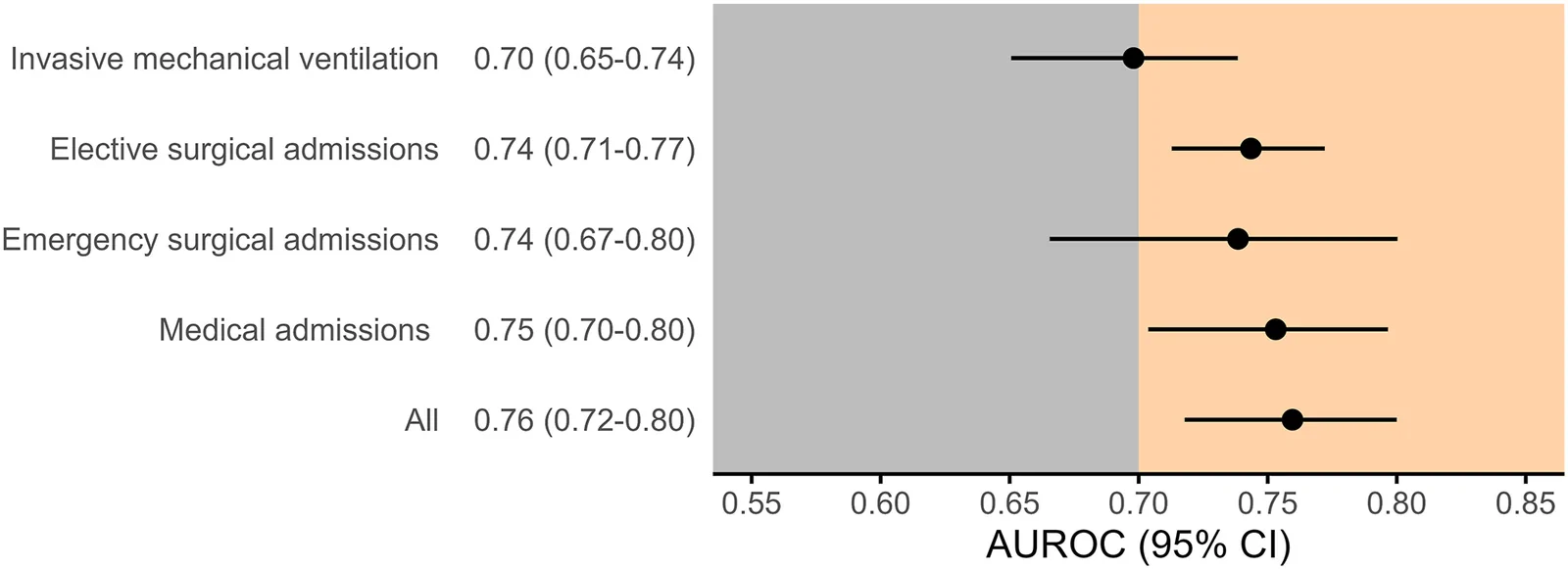
**A forest plot showing the pooled AUROC (95% CI) for each subgroup of the primary (in-hospital mortality) analysis**. Pooled AUROCs were obtained using restricted maximum likelihood estimation for each subgroup. The area shaded orange is above the pre-specified threshold AUROC of 0.7, and the area shaded grey is below this threshold.

Results from the pre-specified subgroup analyses are shown in Supplementary Table 5. For all subgroups, pooled AUROC point estimates were approximately 0.70 or higher (Fig. 3). The worst discrimination was observed in the invasively ventilated subgroup (AUROC 0.70 [0.65–0.74]). Score performance in each subgroup was either comparable to or marginally worse than in the total study population.

#### 3.2.3 Sensitivity analyses

A complete case analysis and an analysis excluding all inward and outward interhospital transfers both showed comparable results to the primary analysis (Supplementary Table 6). Both sensitivity analyses yielded pooled AUROCs of 0.76 (0.72–0.80).

### 3.3 ICU mortality analysis

#### 3.3.1 Main ICU mortality analysis

The study population for the ICU mortality analysis is described in Table 3. In total, 465,790 admissions were included from 546 ICUs in 13 countries. ICU mortality ranged from 5.3% to 56.8%. The median missingness rate for SMS-ICU variables was 0.1% (IQR 0–1.0%) (Table 3). Case-mix for each registry is described in Supplementary Table 7a-l.

**Table 3:** Study population for the ICU mortality analysis.

Registry location		Aus & NZ	Bangladesh	Brazil	Ethiopia	Finland	Ghana	India	Malaysia	Nepal	Netherlands	South Africa	Uruguay
Number of ICUs included		194	1	207	2	25	1	22	4	12	68	4	6
Total ICU admissions during study period		197 713	1334	180 268	564	16 460	209	15 002	2363	7187	70 080	1285	2240
Number of admissions included		183 944	1221	176 866	480	13 573	163	13 690	2106	6326	64 163	1190	2068
Number of exclusions	Total patients excluded	13 768	113	3402	84	2887	46	1312	257	861	5917	95	172
	Age not over 18 years	1582	101	1300	48	998	31	513	95	210	414	52	22
	Readmission to ICU within same hospital encounter	8245	11	0	15	1472	13	401	144	344	3163	18	0
	Interhospital transfer	3685	1	2102	21	416	2	281	18	307	2340	25	150
	Missing outcome	247	0	0	0	26	0	117	0	0	0	0	0
Median patient age (IQR)		66 (52–76)	40 (28–60)	65 (47–78)	40 (28–55)	65 (53–73)	47 (30–61)	60 (47–70)	60 (42–69)	59 (42–73)	66 (55–74)	41 (30–56)	64 (49–74)
Median systolic blood pressure (IQR)		97 (88–108)	110 (90–130)	121 (105–140)	116 (104–130)	90 (82–102)	123 (102–141)	129 (110–140)	129 (114–146)	122 (110–140)	91 (80-105	125 (110–143)	120 (100–142)
Number of male patients (% of total)		103 444 (56.2)	622 (50.9)	85 743 (48.5)	256 (53.3)	8632 (63.6)	73 (44.8)	8123 (59.3)	1175 (55.8)	3572 (56.5)	42 839 (61.1)	758 (63.7)	1225 (59.2)
Number of patients admitted after emergency surgery (% of total)		29 006 (15.8)	316 (25.9)	12 602 (7.1)	95 (19.8)	2441 (18.0)	43 (26.4)	744 (5.4)	248 (11.8)	436 (6.9)	7369 (11.5)	225 (18.9)	224 (10.8)
Number of patients admitted after elective surgery (% of total)		69 651 (37.9)	239 (19.6)	31 899 (18.0)	80 (16.7)	3548 (26.2)	69 (42.3)	2743 (20.0)	368 (17.5)	929 (14.7)	22 615 (35.2)	611 (51.3)	423 (20.4)
Number of patients receiving infused vasopressors/inotropes (% of total)		70 665 (38.4)	300 (24.6)	25 473 (14.4)	105 (21.9)	8231 (60.8)	51 (31.3)	2621 (19.1)	925 (43.9)	1047 (16.6)	31 223 (48.7)	282 (23.7)	461 (22.3)
Number of patients receiving NIV/CPAP (% of total)		18 926 (10.3)	5 (0.41)	20 583 (11.6)	12 (2.5)	1050 (7.1)	1 (0.6)	1105 (8.1)	279 (13.2)	288 (4.6)	NA^a^	101 (8.5)	NA
Number of patients receiving invasive mechanical ventilation (% of total)		55 797 (30.3)	368 (30.1)	4889 (2.8)	331 (69.0)	6101 (45.0)	116 (71.2)	3177 (23.2)	1174 (55.7)	1321 (20.9)	NA	837 (70.3)	718 (34.7)
Number of patients receiving RRT (% of total)		7069 (3.8)	6 (0.5)	14 297 (8.1)	38 (7.9)	638 (4.7)	3 (1.8)	468 (3.4)	163 (7.7)	188 (3.0)	2172 (3.4)	12 (1.0)	65 (3.1)
Total number of ICU deaths (% of total)		9810 (5.3)	694 (56.8)	12 602 (7.1)	160 (33.3)	1008 (7.4)	50 (30.7)	1263 (9.2)	297 (14.1)	815 (12.9)	8384 (13.1)	143 (12.0)	355 (17.2)
Prevalence of missing data for study variables	Blood pressure (% of total)	0 (0)	1 (0.1)	2549 (1.4)	5 (1.0)	187 (1.4)	3 (1.8)	66 (0.5)	0 (0)	11 (0.2)	482 (0.8)	18 (1.5)	84 (4.1)
	Metastasis/haematological malignancy (% of total)	0 (0)	0 (0)	81 (0.0)	0 (0)	669 (4.9)	0 (0)	0 (0)	0 (0)	0 (0)	0 (0)	0 (0)	3 (0.1)
	Vasopressor/inotrope use (% of total)	11 795 (6.4)	1 (0.1)	204 (0.1)	5 (1.0)	0 (0)	3 (1.8)	66 (0.5)	0 (0)	11 (0.2)	0 (0)	18 (1.5)	2 (0.1)
	Respiratory support (% of total)	0 (0)	1 (0.1)	204 (0.1)	5 (1.0)	1528 (11.3)	3 (1.8)	66 (0.5)	0 (0)	11 (0.2)	0 (0)	18 (1.5)	2 (0.1)
	Renal replacement therapy (% of total)	13 084 (7.1)	1 (0.1)	204 (0.1)	5 (1.0)	0 (0)	3 (1.8)	66 (0.5)	0 (0)	11 (0.2)	36 195 (56.4)^b^	18 (1.5)	2 (0.1)
	Admission type (% of total)	0 (0)	0 (0)	0 (0)	0 (0)	29 (0.2)	0 (0)	0 (0)	0 (0)	0 (0)	201 (0.3)	0 (0)	0 (0)

a ‘NA’ refers to data that are not collected by the relevant registry. The Netherlands registry does not report mode of ventilatory support (*i.e*. invasive versus non-invasive).

b Renal replacement therapy (RRT) data are only reported by a subset of ICUs in the Netherlands. In the analysis, RRT data were used where available and modal imputation was applied where not available.

**Fig. 4:**
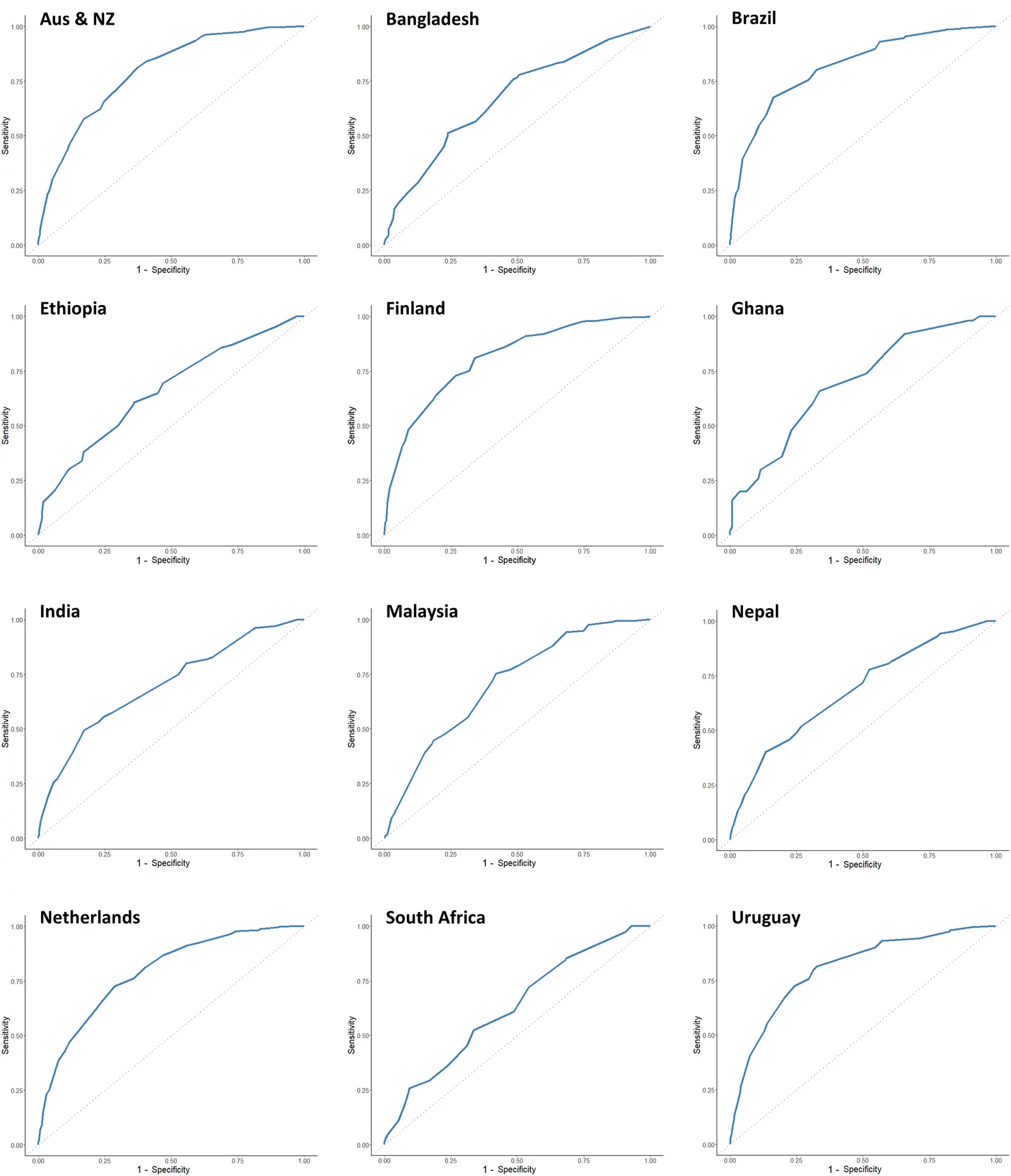
**ROC curves (solid blue) presented by registry for the ICU mortality analysis**. Sensitivity is plotted on the y axis, against (1 – specificity) on the x axis.

ROC curves are shown in Figure 4. AUROCs varied from 0.63 to 0.82, with the pre-specified threshold of 0.70 met in eight of 12 registries (Table 4). The pooled AUROC was 0.73 (0.69–0.77).

**Table 4:** Results from the secondary (ICU mortality) analysis.

	Aus & NZ	Bangladesh	Brazil	Ethiopia	Finland	Ghana	India	Malaysia	Nepal	Netherlands	South Africa	Uruguay
AUROC (95% CI)^a^	0.82 (0.81–0.82)	0.67 (0.64–0.7)	0.82 (0.81–0.82)	0.66 (0.61–0.71)	0.81 (0.79–0.82)	0.70 (0.61–0.78)	0.70 (0.68–0.72)	0.71 (0.68–0.74)	0.68 (0.66–0.7)	0.79 (0.78–0.79)	0.63 (0.58–0.67)	0.80 (0.78–0.83)
Calibration slope^b^	1.0116	0.542	1.0473	0.5702	1.0508	0.7143	0.7067	0.6278	0.6131	0.9225	0.4626	0.8986
Calibration intercept^c^	−1.691	1.8638	−0.8062	0.7306	−1.6543	0.8963	−0.8969	−0.9466	−0.6197	−1.5156	−0.8046	−0.2594
O/E ratio (95% CI)^d^	0.28 (0.27–0.29)	6.18 (6.02–6.35)	0.53 (0.52–0.54)	3.19 (2.96–3.45)	0.31 (0.28–0.33)	2.76 (2.39–3.19)	0.71 (0.67–0.75)	0.66 (0.59–0.74)	1.03 (0.97–1.09)	0.36 (0.35–0.37)	1.34 (1.18–1.53)	0.91 (0.83–0.99)
Brier score^e^	0.0775	0.4571	0.0703	0.2573	0.1019	0.2289	0.0822	0.127	0.107	0.1185	0.1055	0.1158

a AUROC is the area under the receiver operating characteristic.

b Calibration slope and intercept refer to properties of the logistic calibration function. The calibration slope indicates the degree of proportional agreement between predicted and observed outcomes; an ideal calibration curve has a calibration slope of 1.

c The calibration intercept quantifies systematic prediction bias, giving an assessment of calibration-in-the-large. An ideal calibration curve has an intercept of 0.

d The O/E ratio is the ratio of observed to expected events, equivalent to the standardised mortality ratio.

e Brier score ranges from 0 to 1, with values closer to 0 representing better model fit.

**Fig. 5:**
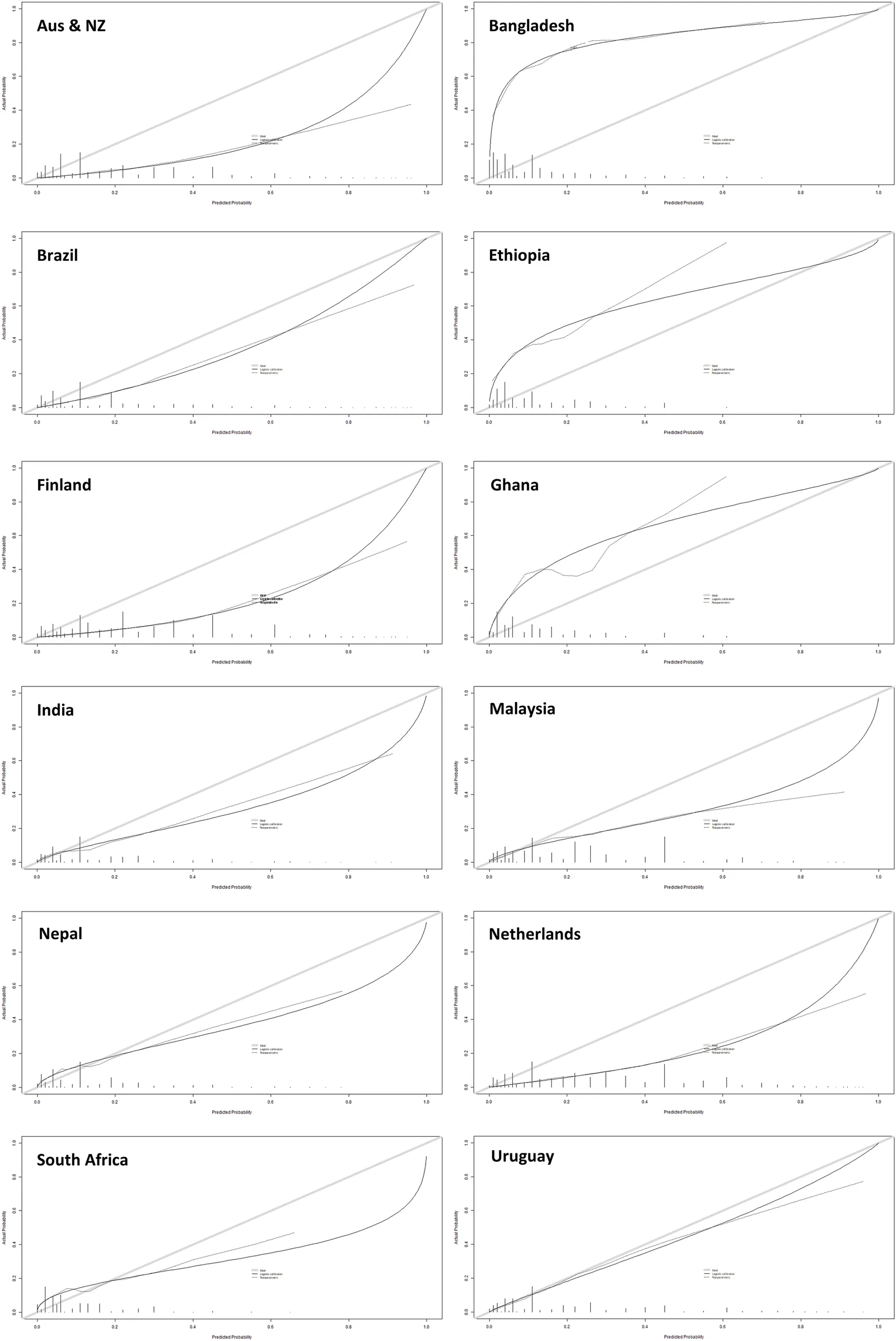
**Calibration curves for ICU mortality analysis**. Observed probability (y-axis) is plotted against predicted probability (x-axis). Logistic calibration curves (solid black) and nonparametric smoothed (dotted) calibration curves are plotted.

There was extensive variation in calibration between registries (Figure 5, Table 4). O/E ratios ranged from 0.28 to 6.18 (Table 4) .

Overall model fit is quantified by Brier scores ranging from 0.070 to 0.457 (Table 4).

#### 3.3.2 Sub-group ICU mortality analysis

**Fig. 6:**
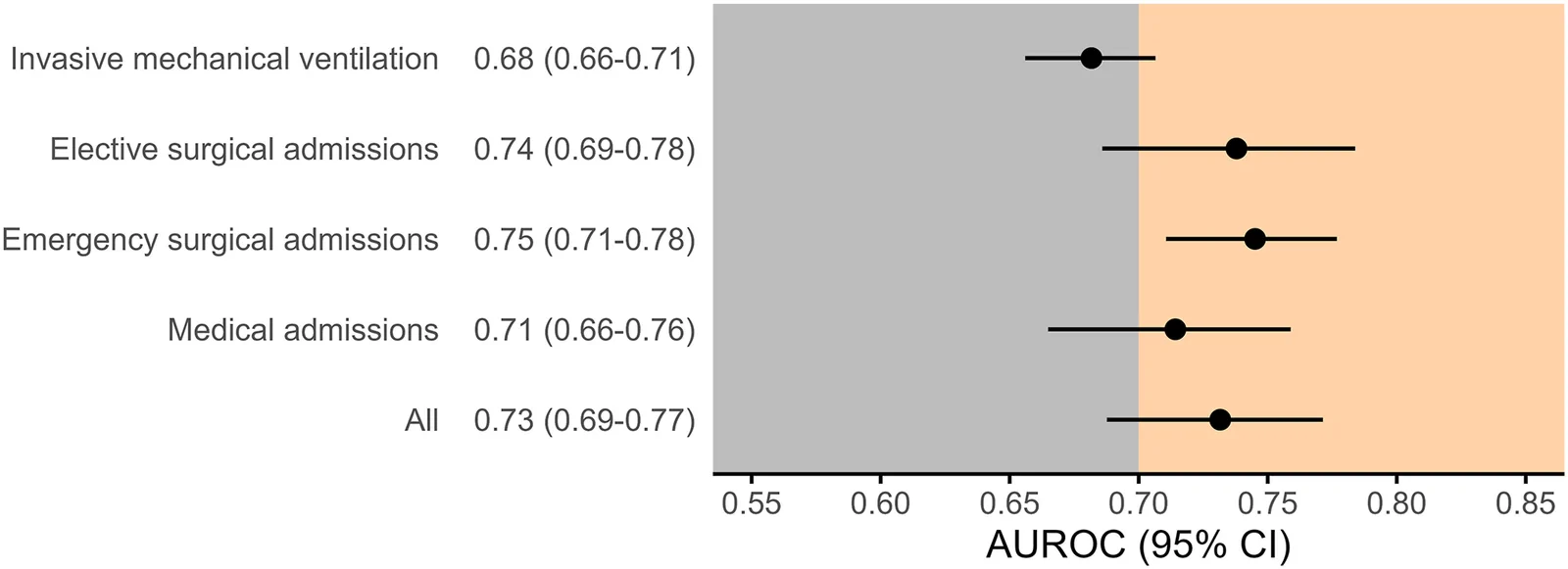
**A forest plot showing the pooled AUROC (95% CI) for each subgroup of the ICU mortality analysis**. Pooled AUROCs were obtained using restricted maximum likelihood estimation for each subgroup. The area shaded orange is above the pre-specified threshold AUROC of 0.7, and the area shaded grey is below this threshold.

Supplementary Table 8 shows results from the pre-specified subgroup analyses, with pooled AUROCs presented in Fig. 6. The worst discrimination was observed in the invasively ventilated subgroup (AUROC 0.68 [0.66–0.71]). All other subgroups had pooled AUROC point estimates above 0.70. As in the primary analysis, score performance was typically comparable or marginally worse than in the total study population.

#### 3.3.3 Sensitivity analyses

The complete case analysis and exclusion of all interhospital transfers yielded comparable results to those described above (Supplementary Table 9). The pooled AUROCs were 0.72 (0.67–0.76) and 0.73 (0.69–0.77), respectively.

## 4 Discussion

### 4.1 Main findings and implications

This study aimed to evaluate the suitability of SMS-ICU for use in critical care registries across multiple continents. Importantly, the prevalence of missing data for SMS-ICU variables was mostly low, suggesting that application to a diverse range of settings is feasible. This is because SMS-ICU uses a subset of variables already widely collected by registries.

Overall, discrimination was acceptable, with a pooled AUROC of 0.76 (0.72–0.80) in the primary analysis. This suggests that SMS-ICU could be appropriately used as an index of illness severity in international critical care populations. This could strengthen collaborative international research and facilitate the synthesis of international results in systematic reviews and meta-analyses. Better discrimination can be achieved using additional variables or more complex models;^28^^,^^29^ however, use of such models across a diverse range of settings would not be feasible.

The secondary analysis revealed that discrimination was poorer in African and Asian settings (Table 4). The observed discriminatory performance is consistent with previous studies of other scores.^7^^,^^30^ One explanation for this finding could be differences in case-mix between settings. Alternatively, in some contexts, the first 24 h of an ICU admission may be less informative regarding a patient's overall prognosis. For example, this may be the case if ICU admission typically occurs later in a patient's illness or follows marked variation in preceding healthcare. In some contexts, there may also be significant heterogeneity, *e.g.* in socioeconomic or nutritional factors, that SMS-ICU does not capture but that nonetheless influences prognosis.

Calibration, using the model derived by Zampieri et al.,^14^ varied substantially between registries. In general, the model overestimated mortality in high-income countries while underestimating mortality in lower-income settings. SMS-ICU was well-calibrated in Brazil and Uruguay, reflecting the location of its customisation.^14^ Calibration and model fit were particularly poor in Bangladesh, Ethiopia and Ghana. However, these registries had relatively small sample sizes, so these results should be interpreted with caution.

Possible reasons for the observed variation in calibration include inter-registry differences in case-mix, ICU performance, healthcare resourcing and health system characteristics. International variation in model calibration is well recognised, having previously motivated the development of regional customisations of the Simplified Acute Physiology Score (SAPS).^31^ Such variation contrasts with the excellent calibration that can be achieved by location-specific prognostic models.^32^^,^^33^

Score performance in subgroup analyses was typically slightly poorer than in the overall population. Because subgroups were defined by SMS-ICU variables, each subgroup analysis effectively removed one component from the score, thus reducing the information available to the model. In addition, the invasively ventilated subgroup is expected to have higher illness severity than the broader study population, making discrimination between individuals inherently more challenging. Therefore, additional variables are likely needed to discriminate between these patients.

Notably, prior to this study, SMS-ICU had not been validated for elective ICU admissions. This study shows that discriminatory performance in elective surgical cases is comparable to that for acute admissions. Therefore, SMS-ICU is applicable to a broader patient population than originally intended.

Taken together, these findings clarify the suitability of SMS-ICU for different international use cases. Given that discriminatory performance was acceptable, SMS-ICU can reasonably be used to describe illness severity. However, international variation in calibration limits its suitability for applications that require reliable absolute risk prediction. For example, these data do not support the routine use of SMS-ICU to estimate expected mortality rates for ICUs across heterogeneous settings.

### 4.2 Strengths and limitations

This study describes the performance of SMS-ICU in a large, diverse, multicontinental population of consecutive patients from critical care registries. This was enabled by its federated design, which obviated the need for international transfer of sensitive patient-level data. There is increasing interest in using such approaches to exploit large real-world datasets while maximising data security.[34], [35], [36] Building on this, the present study demonstrates the value of federated analysis for external validation of a prognostic model.

The handling of some variables required minor adjustments in certain registries to enable the calculation of SMS-ICU scores. This may have adversely affected performance in the affected registries. However, SMS-ICU performed relatively well in settings where this was required.

Although the use of registry datasets enabled real-world evaluation of score performance, there are limitations associated with this data source. Many registries lack full national coverage, so not all are nationally representative.^15^ Individuals admitted to ICUs for palliative care were not excluded, as not all registries identify such patients. Some registries had few contributing ICUs, limiting statistical power and generalisability of the results. This is a particularly important caveat to the findings in African registries. Finally, not all geographical regions were represented in this study.

### 4.3 Conclusion and future directions

In summary, SMS-ICU discrimination and model fit were broadly acceptable within many registries. This suggests that SMS-ICU could reasonably be used as an international index of illness severity. Variation in calibration limits other international uses of SMS-ICU in its current form.

In future, this variation could be addressed by customisation to re-calibrate the model based on contextual variables, such as geographical location, economic indicators or healthcare system characteristics. This could be achieved by developing context-specific equations or by deriving a new multi-level model. However, such work would require the systematic collection of hospital outcomes by a wider range of critical care registries.

## Contributor roles

AT – conceptualisation, formal analysis, methodology, visualisation, writing – original draft.

FF – formal analysis, methodology, software, visualisation, writing – review & editing.

JS, RH, AB, OR – conceptualisation, methodology, resources, supervision, writing – review & editing.

RAMEU, DA, MBMN, GB, DD, SF, BKTV, DP, LP, MR, MS-F, MS, DT – methodology, resources, writing – review & editing; SB, ZD, VI, TP, GT – formal analysis, resources, writing – review & editing.

## Funding

This research did not receive any specific grant from funding agencies in the public, commercial, or not-for-profit sectors.

## Declaration of competing interest

The authors declare the following financial interests/personal relationships which may be considered as potential competing interests: Jorge IF Salluh reports a relationship with Coordenação de Aperfeiçoamento de Pessoal de Nível Superior Brasil (CAPES) that includes: funding grants. Rashan Haniffa reports a relationship with Wellcome Trust that includes: funding grants. Rashan Haniffa reports a relationship with National Institute for Health and Care Research that includes: funding grants. Rashan Haniffa reports a relationship with Minderoo Foundation Limited that includes: funding grants. Abigail Beane reports a relationship with Wellcome Trust that includes: funding grants. Otavio Ranzani reports a relationship with Spanish Ministry of Science, Innovation and Universities that includes: funding grants. Otavio Ranzani reports a relationship with European Social Fund Plus that includes: funding grants. Dave Dongelmans is unpaid chair of the Dutch ICU registry: National Intensive Care Evaluation (NICE). David Pilcher sits on the editorial boards of Critical Care Science and Critical Care and Resuscitation. David Pilcher is also the chair of the ANZICS CORE registry (honorary unpaid position). Given his role as editor, David Pilcher had no involvement in the peer review of this article and had no access to information regarding its peer review. Full responsibility for the editorial process for this article was delegated to another journal editor. Abigail Beane is an unpaid WHO adviser to the Acute Care Action Network. If there are other authors, they declare that they have no known competing financial interests or personal relationships that could have appeared to influence the work reported in this paper.
