# Supplementary material for: Evaluation of the SMS-ICU score for multicontinental use: A federated external validation study using critical care registry data
Source: Crit Care Resusc. 2026 Aug 13;28(3):100206. doi: 10.1016/j.ccrj.2026.100206 (PMC13499179; doi:10.1016/j.ccrj.2026.100206)
Supplement: Supplementary file 1 [file mmc1.docx]

**Supplementary methods**

*Local adaptations to original variable handling*

In Australia & New Zealand and Finland, data regarding vasopressor/inotrope use and renal replacement therapy (RRT) were available only for the duration of an ICU stay, not specifically for the first 24 hours. Given the lack of proven benefit to early RRT and its decreasing rate of initiation over time, patients who received RRT were not assumed to have received it within 24 hours of admission.^1–3^ Therefore, no patients were allocated points for RRT use in these two registries. In contrast, vasopressors/inotropes are commonly administered early in an ICU admission; therefore, patients who received these treatments were allocated the corresponding points.^4,5^

**References**

1.	Li X, Liu C, Mao Z, Li Q, Zhou F. Timing of renal replacement therapy initiation for acute kidney injury in critically ill patients: a systematic review of randomized clinical trials with meta-analysis and trial sequential analysis. Crit Care. 2021 Dec 1;25(1).

2.	Ziegler J, Morley K, Pilcher D, Bellomo R, Soares M, Salluh JIF, et al. Epidemiology of Renal Replacement Therapy for Critically Ill Patients across Seven Health Jurisdictions. Am J Nephrol. 2024 Oct 1;55(5):539–50.

3.	Peerapornratana S, Mahamitra N, Srisawat N. 112 OUTCOMES OF RENAL REPLACEMENT THERAPY IN INTENSIVE CARE UNITS IN THAILAND. Kidney Int Rep [Internet]. 2017 Aug 1 [cited 2025 Sep 24];2(4):S16. Available from: https://www.sciencedirect.com/science/article/pii/S2468024917302243?utm_source=chatgpt.com

4.	Ammar MA, Ammar AA, Wieruszewski PM, Bissell BD, T. Long M, Albert L, et al. Timing of vasoactive agents and corticosteroid initiation in septic shock. Vol. 12, Annals of Intensive Care. Springer Science and Business Media Deutschland GmbH; 2022.

5.	Viglianti EM, Bagshaw SM, Bellomo R, McPeake J, Molling DJ, Wang XQ, et al. Late Vasopressor Administration in Patients in the ICU: A Retrospective Cohort Study. Chest [Internet]. 2020 Aug 1 [cited 2025 Sep 24];158(2):571–8. Available from: https://www.sciencedirect.com/science/article/pii/S0012369220305912
