# Supplementary material for: Evaluation of the SMS-ICU score for multicontinental use: A federated external validation study using critical care registry data
Source: Crit Care Resusc. 2026 Aug 13;28(3):100206. doi: 10.1016/j.ccrj.2026.100206 (PMC13499179; doi:10.1016/j.ccrj.2026.100206)
Supplement: Supplementary file 2 [file mmc2.docx]

**Supplementary Tables and Figures**

**Contents**

Supplementary Table 1: SMS-ICU component variables

Supplementary Table 2: Summary description of participating registries

Supplementary Table 3: description of the original study populations for development, customisation and validation of the SMS-ICU score, presented for comparison

Supplementary Table 4 (A-G): case-mix by registry for the in-hospital mortality analysis.

Supplementary Table 5: Results from subgroup analyses of the in-hospital mortality validation study presented for each registry

Supplementary Table 6: Results from in-hospital mortality sensitivity analyses.

Supplementary Tables 7 (A-L): case-mix by registry for the ICU mortality analysis.

Supplementary Table 8: Results from subgroup analyses in the ICU mortality analysis

Supplementary Table 9: Results from ICU mortality sensitivity analyses

Supplementary Figure 1: Participant flow diagram across all registries included in the primary analysis

Supplementary Figure 2: Participant flow diagram across all registries for the secondary (ICU mortality) analysis

**Supplementary Table 1: SMS-ICU component variables**

| **Variable** | **Points^a^** |
| --- | --- |
| Age (years):  ≤39  40-59  60-79  ≥80 | 0  5  10  13 |
| Systolic blood pressure (mmHg):  ≤49  50-69  70-89  ≥90 | 6  5  3  0 |
| Admission type:  Medical  Emergency surgical | 3  0 |
| Haematological malignancy or metastatic cancer | 7 |
| Vasopressor or inotrope use^b^ | 4 |
| Respiratory support^c^ | 5 |
| Renal replacement therapy^d^ | 4 |

^a^allocated as per Granholm *et al.* (2018). The score is calculated using the highest scoring values recorded during the first 24 hours of an admission to the ICU.

^b^refers to continuous use rather than isolated bolus doses

^c^refers to both invasive and non-invasive ventilation, but excludes use of continuous positive airway pressure for exercises alone

^d^refers to continuous or intermittent RRT for both acute and chronic indications

**Supplementary Table 2: Summary description of participating registries**

| Registry name | | **ANZICS APD^a^** | **Bangladesh** | **Brazil** | **Ethiopia** | **FICC^b^** | **Ghana** | **IRIS^c^** | **Malaysia** | **NICR^d^** | **NICE^e^** | **South Africa** | **Uruguay** |
| --- | --- | --- | --- | --- | --- | --- | --- | --- | --- | --- | --- | --- | --- |
| Countries included in registry | | Australia & New Zealand | Bangladesh | Brazil | Ethiopia | Finland | Ghana | India | Malaysia | Nepal | Netherlands | South Africa | Uruguay |
| Total no. of contributing ICUs during study period | | 194 | 2 | 207 | 4 | 25 | 1 | 33 | 5 | 21 | 68 | 7 | 6 |
| Total no. of ICU beds covered during study period | | 2655 | 36 | 3916 | 45 | 247 | 6 | 633 | 57 | 315 | 1115 | 43 | 84 |
| No. of ICUs by type | Mixed or general | 191 | - | 154 | 4 | 22 | 1 | 21 | 4 | 15 | 68 | 3 | 6 |
|  | Medical | - | 2 | 12 | - | - | - | 7 | - | 4 | - | 1 | - |
|  | Surgical | - | - | 15 | - | - | - | 2 | 1 | 1 | - | 1 | - |
|  | Cardiothoracic | 3 | - | - | - | 2 | - | 1 | - | - | - | 1 | - |
|  | Liver | - | - | - | - | - | - | 1 | - | 1 | - | - | - |
|  | Neurological | - | - | 13 | - | 1 | - | 1 | - | - | - | 1 | - |
| Percentage of ICUs that are publicly funded (%) | | 64 | 50 | 30.4 | 100 | 100 | 100 | 18.2 | 100 | 42.9 | 100 | 100 | 16.7 |
| Registry commencement year | | 1993 | 2020 | 2009 | 2020 | 1994 | 2021 | 2019 | 2019 | 2019 | 1996 | 2021 | 2016 |
| Automated data extraction used for vital signs^f^ | | Y | N | Y | N | Y | N | N | N | N | Y | N | Y |
| Existing score used to benchmark mortality | | ANZROD | APACHE II | SAPS3 | APACHE II | SAPS2 | APACHE II | APACHE II | APACHE II | APACHE II | APACHE IV | APACHE II | SAPS3 |

^a^Australian and New Zealand Intensive Care Society Adult Patient Database

^b^Finnish Intensive Care Consortium

^c^Indian Registry of Intensive Care

^d^Nepal Intensive Care Research Foundation

^e^National Intensive Care Evaluation

^f^This is reported per registry. Use of automated data entry methods may vary across constituent sites.

**Supplementary Table 3: description of the original study populations for development, customisation and validation of the SMS-ICU score, presented for comparison**

|  | | **Development** | **Customisation** | **Validation** |
| --- | --- | --- | --- | --- |
| Setting | | Trial datasets | Brazil | Brazil |
| Total number of participants included | | 4086 | 43017 | 313365 |
| Median patient age (IQR) | | 66 (55-75) | 67 (50-80) | 64 (45-80) |
| Median systolic blood pressure (IQR) | | 86 (75-100) | 125 (107-144) | 128 (111-145) |
| Number of male patients (% of total) | | 2385 (58.4) | NA | NA |
| Number of patients admitted after emergency surgery (% of total) | | 1290 (31.6) | 3174 (7.4) | 12389 (4.0) |
| Number of patients admitted after elective surgery (% of total) | | 0 | 0 | 0 |
| Number of patients receiving infused vasopressors/inotropes (% of total) | | 2583  (63.2) | 5559  (12.9) | 17312  (5.5) |
| Number of patients receiving NIV (% of total) | | NA | 4623 (10.8) | 22977 (7.3) |
| Number of patients receiving invasive mechanical ventilation (% of total) | | NA | 6445 (15.0) | 15405 (4.9) |
| Number of patients receiving respiratory support (% of total) | | 2577 (63.1) | 11068 (25.7) | 38382 (12.2) |
| Number of patients receiving RRT (% of total) | | 376 (9.2) | 1404 (3.3) | 4849 (1.5) |
| Total number of in-hospital deaths (% of total) | | NA | 7743 (18.0) | 28924 (9.2) |
| Total number of deaths at 90 days (% of total) | | 1403 (34.3) | NA | NA |
| Prevalence of missing data for study variables | Blood pressure (% of total) | 22 (0.5) | 3% | 1.9% |
|  | Metastasis/haematological malignancy (% of total) | 0 | 0 | 0 |
|  | Vasopressor/inotrope use (% of total) | 0 | 0 | 0.7% |
|  | Respiratory support (% of total) | 0 | 0 |  |
|  | Renal replacement therapy (% of total) | 0 | 0 |  |
|  | Admission type (% of total) | 0 | 0 | 0 |

**Supplementary Table 4 (A-G): case-mix by registry for the in-hospital mortality analysis.** Admission diagnosis is grouped according to either APACHE II diagnostic categories or each registry’s own classification system.

A.

|  | **Aus & NZ** |
| --- | --- |
| Other* (% of total) | 36297 (19.8) |
| Gastrointestinal (% of total) | 28275 (15.4) |
| Respiratory disorders (% of total) | 27501 (15.0) |
| Cardiac disease (% of total) | 25812 (14.1) |
| Neurological disorders (% of total) | 24078 (13.1) |
| Post-cardiac surgery (% of total) | 17685 (9.6) |
| Sepsis and septic shock (% of total) | 15634 (8.5) |
| Trauma (% of total) | 8162 (4.4) |

*The most common diagnoses within the ‘other’ category are shown below.

| **Most common diagnosis within 'other' category** | |
| --- | --- |
| Post-op: orthopaedic surgery | 8910 (24.5%) |
| Drug overdose | 5963 (16.4%) |
| Other metabolic disorders | 3235 (8.9%) |

B.

|  | **Brazil** |
| --- | --- |
| Sepsis and septic shock (% of total) | 42264 (23.9) |
| Postoperative care (% of total) | 38513 (21.8) |
| Cardiac disease (% of total) | 26738 (15.1) |
| Neurological disorders (% of total) | 20511 (11.6) |
| Metabolic/renal/GI/hypovolaemic shock (% of total) | 19905 (11.3) |
| Respiratory disorders (% of total) | 6363 (3.6) |
| Trauma and poisoning (% of total) | 6226 (3.5) |
| Post-cardiac arrest (% of total) | 774 (0.4) |

C.

|  | **Finland** |
| --- | --- |
| Postoperative care (% of total) | 5938 (43.7) |
| Metabolic/renal/GI/hypovolaemic shock (non-operative) (% of total) | 1776 (13.1) |
| Respiratory disorders (non-operative) (% of total) | 1512 (11.1) |
| Trauma and poisoning (non-operative) (% of total) | 1087 (8.0) |
| Neurological disorders (non-operative) % of total) | 979 (7.2) |
| Cardiac disease (non-operative) (% of total) | 896 (6.6) |
| Sepsis and septic shock (% of total) | 635 (4.7) |
| Post-cardiac arrest (% of total) | 582 (4.3) |

D.

|  | **India** |
| --- | --- |
| Cardiovascular (non-operative) (% of total) | 1367 (20.7) |
| Respiratory (non-operative) (% of total) | 1192 (18.0) |
| Neurologic (non-operative) (% of total) | 943 (14.3) |
| Gastrointestinal (non-operative) (% of total) | 612 (9.25) |
| Gastrointestinal (operative) (% of total) | 421 (6.4) |
| Genitourinary (non-operative) (% of total) | 403 (6.1) |
| Metabolic/Endocrine (non-operative) (% of total) | 260 (3.9) |
| Haematology (non-operative) (% of total) | 250 (3.8) |
| Musculoskeletal/Skin (non-operative) (% of total) | 143 (2.2) |
| Trauma (non-operative) (% of total) | 110 (1.7) |

E.

|  | **Nepal** |
| --- | --- |
| Respiratory (non-operative) (% of total) | 142 (22.9) |
| Cardiovascular (non-operative) (% of total) | 130 (20.9) |
| Neurologic (non-operative) (% of total) | 96 (15.5) |
| Gastrointestinal (non-operative) (% of total) | 62 (10.0) |
| Cardiovascular (operative) (% of total) | 36 (5.8) |
| Metabolic/Endocrine (non-operative) (% of total) | 33 (5.3) |
| Genitourinary (non-operative) (% of total) | 23 (3.7) |
| Gastrointestinal (operative) (% of total) | 16 (2.6) |
| Genitourinary (operative) (% of total) | 9 (1.4) |
| Trauma (non-operative) (% of total) | 8 (1.3) |

F.

|  | **Netherlands** |
| --- | --- |
| Cardiovascular (N, % of total) | 30778 (44.0) |
| Respiratory (N, % of total) | 12743 (18.2) |
| Neurological (N, % of total) | 9955 (14.2) |
| GI (N, % of total) | 7125 (10.2) |
| Trauma (N, % of total) | 3285 (4.7) |
| Genito-urinary (N, % of total) | 2159 (3.1) |
| Metabolic/endocrine (N, % of total) | 1678 (2.4) |
| Musculoskeletal/skin (N, % of total) | 1393 (2.0) |
| Haematological (N, % of total) | 453 (0.7) |
| Transplant (N, % of total) | 449 (0.6) |

G.

|  | **Uruguay** |
| --- | --- |
| Postoperative care (% of total) | 558 (27.0) |
| Sepsis and septic shock (% of total) | 306 (14.8) |
| Neurological disorders (% of total) | 289 (14.0) |
| Cardiac disease (% of total) | 277 (13.4) |
| Trauma and poisoning (% of total) | 213 (10.3) |
| Metabolic/renal/GI/hypovolaemic shock (% of total) | 152 (7.4) |
| Respiratory disorders (% of total) | 141 (6.8) |
| Post-cardiac arrest (% of total) | 28 (1.4) |

**Supplementary Table 5: Results from subgroup analyses of the in-hospital mortality validation study presented for each registry**

|  | **Aus & NZ** | **Brazil** | **Finland** | **India** | **Nepal** | **Netherlands** | **Uruguay** |
| --- | --- | --- | --- | --- | --- | --- | --- |
|  | **Medical admissions** | | | | | | |
| AUROC (95% CI) | 0.76 (0.76-0.77) | 0.8 (0.79-0.8) | 0.79 (0.77-0.8) | 0.64 (0.62-0.66) | 0.72 (0.66-0.79) | 0.78 (0.78-0.79) | 0.76 (0.73-0.79) |
| Calibration slope | 0.8165 | 1.0131 | 0.8888 | 0.505 | 1.0296 | 0.8151 | 0.7983 |
| Calibration intercept | -0.8755 | -0.2339 | -0.6528 | -0.6174 | -0.0624 | -0.6648 | 0.0093 |
| O/E ratio (95% CI) | 0.61 (0.6-0.62) | 0.84 (0.83-0.85) | 0.67 (0.64-0.71) | 1.13 (1.08-1.19) | 0.92 (0.75-1.14) | 0.72 (0.7-0.73) | 1.14 (1.06-1.23) |
| Brier score | 0.1223 | 0.1004 | 0.1318 | 0.1361 | 0.1021 | 0.1422 | 0.1603 |
|  | **Emergency surgical admissions** | | | | | | |
| AUROC (95% CI) | 0.76 (0.75-0.76) | 0.79 (0.78-0.8) | 0.75 (0.71-0.78) | 0.47 (0.34-0.6) | 0.79 (NA-NA) | 0.7 (0.68-0.72) | 0.79 (0.7-0.87) |
| Calibration slope | 0.8057 | 0.9757 | 0.8041 | -0.1384 | 0.7482 | 0.6167 | 0.79 |
| Calibration intercept | -1.3876 | 0.305 | -1.0352 | -2.1145 | -0.6606 | -1.0397 | 0.1664 |
| O/E ratio (95% CI) | 0.4 (0.38-0.42) | 1.28 (1.24-1.32) | 0.48 (0.42-0.55) | 1.48 (0.99-2.2) | 0.92 (0.14-6.09) | 0.59 (0.56-0.62) | 1.38 (1.12-1.7) |
| Brier score | 0.0847 | 0.1096 | 0.1088 | 0.1509 | 0.0647 | 0.152 | 0.126 |
|  | **Elective surgical admissions** | | | | | | |
| AUROC (95% CI) | 0.75 (0.73-0.77) | 0.77 (0.76-0.78) | 0.74 (0.67-0.81) | 0.73 (0.66-0.79) | 0.85 (0.73-0.98) | 0.69 (0.67-0.72) | 0.72 (0.61-0.83) |
| Calibration slope | 0.7982 | 0.9079 | 0.8791 | 0.9044 | 1.1497 | 0.6524 | 0.8179 |
| Calibration intercept | -3.4315 | -0.933 | -3.2591 | -0.2289 | 0.0168 | -3.1698 | -0.6788 |
| O/E ratio (95% CI) | 0.06 (0.04-0.08) | 0.53 (0.5-0.56) | 0.06 (0.02-0.16) | 0.98 (0.8-1.21) | 0.75 (0.26-2.16) | 0.09 (0.07-0.11) | 0.77 (0.53-1.13) |
| Brier score | 0.0455 | 0.0478 | 0.0758 | 0.0629 | 0.041 | 0.0993 | 0.0594 |
|  | **Patients receiving invasive mechanical ventilation** | | | | | | |
| AUROC (95% CI) | 0.71 (0.7-0.72) | 0.7 (0.69-0.71) | 0.75 (0.73-0.77) | 0.58 (0.55-0.61) | 0.63 (0.48-0.78) | NA^a^ | 0.73 (0.7-0.77) |
| Calibration slope | 0.8086 | 0.6543 | 0.9085 | 0.2832 | 0.4851 | NA | 0.6817 |
| Calibration intercept | -1.2869 | 0.4523 | -0.9997 | -0.3334 | 0.1349 | NA | 0.1584 |
| O/E ratio (95% CI) | 0.44 (0.43-0.45) | 1.38 (1.36-1.39) | 0.53 (0.49-0.56) | 1.41 (1.32-1.5) | 1.47 (1.13-1.92) | NA | 1.22 (1.13-1.32) |
| Brier score | 0.1483 | 0.2393 | 0.147 | 0.2312 | 0.2374 | NA | 0.2082 |

^a^ ‘NA’ refers to data that are not collected by the registry concerned.

**Supplementary Table 6: Results from in-hospital mortality sensitivity analyses.**

|  | **Aus & NZ** | **Brazil** | **Finland** | **India** | **Nepal** | **Netherlands** | **Uruguay** |
| --- | --- | --- | --- | --- | --- | --- | --- |
|  | **Complete case analysis** | | | | | | |
| AUROC (95% CI) | 0.79 (0.79-0.8) | 0.8 (0.8-0.81) | 0.79 (0.77-0.8) | 0.66 (0.64-0.68) | 0.75 (0.69-0.8) | 0.75 (0.75-0.76) | 0.78 (0.76-0.81) |
| Calibration slope | 0.9207 | 1.0134 | 0.9599 | 0.5585 | 1.045 | 0.7855 | 0.8406 |
| Calibration intercept | -1.2495 | -0.2547 | -1.0815 | -0.5765 | -0.0511 | -1.2518 | 2e-04 |
| O/E ratio (95% CI) | 0.42 (0.42-0.43) | 0.83 (0.82-0.84) | 0.49 (0.46-0.51) | 1.12 (1.07-1.18) | 0.91 (0.74-1.12) | 0.47 (0.45-0.49) | 1.13 (1.05-1.21) |
| Brier score | 0.0897 | 0.0911 | 0.1166 | 0.1248 | 0.0921 | 0.1366 | 0.1367 |
|  | **Excluding all inter-hospital transfers** | | | | | | |
| AUROC (95% CI) | 0.79 (0.79-0.8) | 0.8 (0.8-0.81) | 0.78 (0.77-0.79) | 0.66 (0.64-0.68) | 0.75 (0.69-0.8) | 0.76 (0.76-0.77) | 0.78 (0.76-0.81) |
| Calibration slope | 0.9269 | 1.0076 | 0.9333 | 0.5565 | 1.046 | 0.8298 | 0.8355 |
| Calibration intercept | -1.2636 | -0.295 | -1.0509 | -0.5868 | -0.0627 | -1.2021 | -0.0197 |
| O/E ratio (95% CI) | 0.42 (0.41-0.42) | 0.81 (0.8-0.82) | 0.49 (0.47-0.52) | 1.12 (1.06-1.17) | 0.9 (0.73-1.11) | 0.48 (0.47-0.49) | 1.12 (1.04-1.2) |
| Brier score | 0.0861 | 0.0888 | 0.1129 | 0.1243 | 0.0913 | 0.1267 | 0.1353 |

**Supplementary Tables 7 (A-L): case-mix by registry for the ICU mortality analysis.** Admission diagnosis is grouped according to either APACHE II diagnostic categories or each registry’s own classification system.

A.

|  | **Aus & NZ** |
| --- | --- |
| Other* (% of total) | 36523 (19.9) |
| Gastrointestinal (% of total) | 28358 (15.4) |
| Respiratory disorders (% of total) | 27540 (15.0) |
| Cardiac disease (% of total) | 25841 (14.0) |
| Neurological disorders (% of total) | 24127 (13.1) |
| Post-cardiac surgery (% of total) | 17698 (9.6) |
| Sepsis and septic shock (% of total) | 15667 (8.5) |
| Trauma (% of total) | 8190 (4.5) |

*The most common diagnoses within the ‘other’ category are shown below.

| **Most common diagnoses within 'other' category** | |
| --- | --- |
| Post-op: orthopaedic surgery | 8997 (24.6%) |
| Drug overdose | 5976 (16.4% |
| Other metabolic disorders | 3240 (8.9%) |

B.

|  | **Bangladesh** |
| --- | --- |
| Gastrointestinal (operative) (% of total) | 196 (16.1) |
| Respiratory (non-operative) (% of total) | 180 (14.7) |
| Neurologic (non-operative) (% of total) | 177 (14.5) |
| Cardiovascular (non-operative) (% of total) | 166 (13.6) |
| Genitourinary (operative) (% of total) | 152 (12.5) |
| Neurologic (operative) (% of total) | 92 (7.5) |
| Trauma (non-operative) (% of total) | 42 (3.4) |
| Gastrointestinal (non-operative) (% of total) | 40 (3.3) |
| Genitourinary (non-operative) (% of total) | 36 (3.0) |
| Respiratory (operative) (% of total) | 33 (2.7) |

C.

|  | **Brazil** |
| --- | --- |
| Sepsis and septic shock (% of total) | 42264 (23.9) |
| Postoperative care (% of total) | 38513 (21.8) |
| Cardiac disease (% of total) | 26738 (15.1) |
| Neurological disorders (% of total) | 20511 (11.6) |
| Metabolic/renal/GI/hypovolaemic shock (% of total) | 19905 (11.3) |
| Respiratory disorders (% of total) | 6363 (3.6) |
| Trauma and poisoning (% of total) | 6226 (3.5) |
| Post-cardiac arrest (% of total) | 774 (0.4) |

D.

|  | **Ethiopia** |
| --- | --- |
| Respiratory (non-operative) (% of total) | 160 (33.3) |
| Neurologic (operative) (% of total) | 68 (14.2) |
| Cardiovascular (non-operative) (% of total) | 56 (11.7) |
| Gastrointestinal (operative) (% of total) | 55 (11.5) |
| Trauma (non-operative) (% of total) | 35 (7.3) |
| Neurologic (non-operative) (% of total) | 28 (5.8) |
| Genitourinary (operative) (% of total) | 13 (2.7) |
| Cardiovascular (operative) (% of total) | 10 (2.1) |
| Genitourinary (non-operative) (% of total) | 9 (1.9) |
| Respiratory (operative) (% of total) | 9 (1.9) |

E.

|  | **Finland** |
| --- | --- |
| Postoperative care (% of total) | 5938 (43.8) |
| Metabolic/renal/GI/hypovolaemic shock (non-operative) (% of total) | 1776 (13.1) |
| Respiratory disorders (non-operative) (% of total) | 1512 (11.1) |
| Trauma and poisoning (non-operative) (% of total) | 1087 (8.0) |
| Neurological disorders (non-operative) % of total) | 979 (7.2) |
| Cardiac disease (non-operative) (% of total) | 896 (6.6) |
| Sepsis and septic shock (% of total) | 635 (4.7) |
| Post-cardiac arrest (% of total) | 582 (4.3) |

F.

|  | **Ghana** |
| --- | --- |
| Neurologic (operative) (% of total) | 47 (28.8) |
| Gastrointestinal (operative) (% of total) | 34 (20.9) |
| Respiratory (non-operative) (% of total) | 16 (9.8) |
| Cardiovascular (non-operative) (% of total) | 14 (8.6) |
| Musculoskeletal/Skin (operative) (% of total) | 11 (6.8) |
| Genitourinary (operative) (% of total) | 9 (5.5) |
| Genitourinary (non-operative) (% of total) | 8 (4.9) |
| Neurologic (non-operative) (% of total) | 4 (2.5) |
| Haematology (non-operative) (% of total) | 3 (1.8) |
| Metabolic/Endocrine (non-operative) (% of total) | 3 (1.8) |

G.

|  | **India** |
| --- | --- |
| Cardiovascular (non-operative) (% of total) | 2658 (19.4) |
| Respiratory (non-operative) (% of total) | 2196 (16.0) |
| Neurologic (non-operative) (% of total) | 1928 (14.1) |
| Gastrointestinal (non-operative) (% of total) | 989 (7.2) |
| Gastrointestinal (operative) (% of total) | 830 (6.1) |
| Genitourinary (non-operative) (% of total) | 694 (5.1) |
| Metabolic/Endocrine (non-operative) (% of total) | 531 (3.9) |
| Cardiovascular (operative) (% of total) | 484 (3.5) |
| Neurologic (operative) (% of total) | 478 (3.5) |
| Musculoskeletal/Skin (operative) (% of total) | 355 (2.6) |

H.

|  | **Malaysia** |
| --- | --- |
| Cardiovascular (non-operative) (% of total) | 537 (25.5) |
| Respiratory (non-operative) (% of total) | 437 (20.8) |
| Gastrointestinal (operative) (% of total) | 163 (7.7) |
| Metabolic/Endocrine (non-operative) (% of total) | 131 (6.2) |
| Gastrointestinal (non-operative) (% of total) | 107 (5.1) |
| Neurologic (non-operative) (% of total) | 104 (4.9) |
| Musculoskeletal/Skin (operative) (% of total) | 102 (4.8) |
| Neurologic (operative) (% of total) | 62 (2.9) |
| Genitourinary (operative) (% of total) | 58 (2.8) |
| Trauma (non-operative) (% of total) | 46 (2.2) |

I.

|  | **Nepal** |
| --- | --- |
| Respiratory (non-operative) (% of total) | 1213 (19.2) |
| Neurologic (non-operative) (% of total) | 1098 (17.4) |
| Gastrointestinal (non-operative) (% of total) | 795 (12.6) |
| Cardiovascular (non-operative) (% of total) | 785 (12.4) |
| Neurologic (operative) (% of total) | 386 (6.1) |
| Gastrointestinal (operative) (% of total) | 365 (5.8) |
| Genitourinary (non-operative) (% of total) | 304 (4.8) |
| Trauma (non-operative) (% of total) | 251 (4.0) |
| Metabolic/Endocrine (non-operative) (% of total) | 241 (3.8) |
| Musculoskeletal/Skin (operative) (% of total) | 118 (1.9) |

J.

|  | **Netherlands** |
| --- | --- |
| Cardiovascular (N, % of total) | 30778 (44.0) |
| Respiratory (N, % of total) | 12743 (18.2) |
| Neurological (N, % of total) | 9955 (14.2) |
| GI (N, % of total) | 7125 (10.2) |
| Trauma (N, % of total) | 3285 (4.7) |
| Genito-urinary (N, % of total) | 2159 (3.1) |
| Metabolic/endocrine (N, % of total) | 1678 (2.4) |
| Musculoskeletal/skin (N, % of total) | 1393 (2.0) |
| Haematological (N, % of total) | 453 (0.7) |
| Transplant (N, % of total) | 449 (0.6) |

K.

|  | **South Africa** |
| --- | --- |
| Cardiovascular (operative) | 231 (19.4) |
| Respiratory (operative) | 215 (18.1) |
| Gastrointestinal (operative) | 146 (12.3) |
| Neurologic (operative) | 107 (9.0) |
| Neurologic (non-operative) | 90 (7.6) |
| Trauma (non-operative) | 90 (7.6) |
| Respiratory (non-operative) | 60 (5.0) |
| Gastrointestinal (non-operative) | 30 (2.5) |
| Cardiovascular (non-operative) | 29 (2.4) |
| Genitourinary (non-operative) | 27 (2.3) |

L.

|  | **Uruguay** |
| --- | --- |
| Postoperative care (% of total) | 558 (27.0) |
| Sepsis and septic shock (% of total) | 306 (14.8) |
| Neurological disorders (% of total) | 289 (14.0) |
| Cardiac disease (% of total) | 277 (13.4) |
| Trauma and poisoning (% of total) | 213 (10.3) |
| Metabolic/renal/GI/hypovolaemic shock (% of total) | 152 (7.4) |
| Respiratory disorders (% of total) | 141 (6.8) |
| Post-cardiac arrest (% of total) | 28 (1.4) |

**Supplementary Table 8: Results from subgroup analyses in the ICU mortality study**

|  | **Aus & NZ** | **Bangladesh** | **Brazil** | **Ethiopia** | **Finland** | **Ghana** | **India** | **Malaysia** | **Nepal** | **Netherlands** | **South Africa** | **Uruguay** |
| --- | --- | --- | --- | --- | --- | --- | --- | --- | --- | --- | --- | --- |
|  | **Medical admissions** | | | | | | | | | | | |
| AUROC (95% CI) | 0.78 (0.78-0.79) | 0.67 (0.63-0.71) | 0.81 (0.81-0.82) | 0.62 (0.55-0.68) | 0.8 (0.79-0.82) | 0.63 (0.48-0.78) | 0.67 (0.65-0.69) | 0.68 (0.64-0.71) | 0.66 (0.64-0.69) | 0.8 (0.79-0.81) | 0.62 (0.54-0.69) | 0.77 (0.74-0.8) |
| Calibration slope | 0.874 | 0.5707 | 1.0434 | 0.4438 | 0.9613 | 0.556 | 0.6209 | 0.5575 | 0.5635 | 0.8777 | 0.4746 | 0.825 |
| Calibration intercept | -1.3314 | 2.2997 | -0.8051 | 0.4714 | -1.2999 | 1.1767 | -0.9215 | -0.8657 | -0.6298 | -1.0428 | -0.4226 | -0.2601 |
| O/E ratio (95% CI) | 0.42 (0.41-0.43) | 6.37 (6.17-6.58) | 0.54 (0.53-0.55) | 2.95 (2.69-3.25) | 0.43 (0.39-0.46) | 3.46 (2.89-4.15) | 0.76 (0.72-0.8) | 0.73 (0.65-0.81) | 1.07 (1.01-1.14) | 0.54 (0.53-0.56) | 1.67 (1.41-1.98) | 0.94 (0.86-1.03) |
| Brier score | 0.1077 | 0.53 | 0.0784 | 0.278 | 0.118 | 0.3513 | 0.0982 | 0.1505 | 0.1191 | 0.1298 | 0.147 | 0.1412 |
|  | **Emergency surgical admissions** | | | | | | | | | | | |
| AUROC (95% CI) | 0.78 (0.77-0.79) | 0.65 (0.59-0.71) | 0.8 (0.79-0.81) | 0.73 (0.61-0.86) | 0.77 (0.73-0.81) | 0.8 (0.67-0.94) | 0.71 (0.64-0.77) | 0.67 (0.56-0.78) | 0.73 (0.67-0.8) | 0.72 (0.7-0.73) | 0.64 (0.53-0.74) | 0.81 (0.73-0.9) |
| Calibration slope | 0.8958 | 0.4697 | 1.0038 | 1.0904 | 0.9324 | 1.2459 | 0.667 | 0.5497 | 0.7464 | 0.6731 | 0.476 | 0.8926 |
| Calibration intercept | -1.8563 | 1.3741 | -0.193 | 2.0822 | -1.7123 | 2.666 | -1.03 | -1.2555 | -0.1339 | -1.4163 | -0.7686 | -0.0654 |
| O/E ratio (95% CI) | 0.25 (0.23-0.27) | 6.61 (6.25-7) | 0.86 (0.82-0.91) | 3.83 (3.16-4.63) | 0.27 (0.21-0.35) | 3.71 (2.96-4.64) | 0.74 (0.55-1) | 0.6 (0.39-0.93) | 1.34 (1.09-1.65) | 0.42 (0.39-0.46) | 1.39 (1.03-1.87) | 1.06 (0.81-1.39) |
| Brier score | 0.0729 | 0.3889 | 0.0796 | 0.1906 | 0.0935 | 0.2684 | 0.0621 | 0.0971 | 0.1035 | 0.1384 | 0.1017 | 0.1001 |
|  | **Elective surgical admissions** | | | | | | | | | | | |
| AUROC (95% CI) | 0.87 (0.85-0.9) | 0.63 (0.56-0.7) | 0.8 (0.79-0.82) | 0.62 (0.49-0.76) | 0.84 (0.79-0.88) | 0.61 (0.41-0.8) | 0.75 (0.69-0.81) | 0.7 (0.54-0.86) | 0.69 (0.61-0.76) | 0.81 (0.78-0.83) | 0.6 (0.52-0.67) | 0.79 (0.64-0.93) |
| Calibration slope | 1.3866 | 0.4072 | 1.0282 | 0.5664 | 1.1485 | 0.4062 | 1.0157 | 0.7325 | 0.6823 | 1.1685 | 0.3709 | 1.011 |
| Calibration intercept | -4.0797 | 0.8774 | -1.4311 | 0.7852 | -4.358 | -0.8621 | -1.1959 | -1.9459 | -1.4077 | -3.6857 | -1.329 | -0.8774 |
| O/E ratio (95% CI) | 0.02 (0.01-0.05) | 5.04 (4.66-5.44) | 0.28 (0.25-0.32) | 4.03 (3.37-4.83) | 0.02 (0-0.33) | 1.37 (0.83-2.25) | 0.33 (0.23-0.48) | 0.26 (0.11-0.65) | 0.51 (0.35-0.77) | 0.04 (0.03-0.07) | 1.08 (0.86-1.37) | 0.47 (0.25-0.88) |
| Brier score | 0.0426 | 0.3439 | 0.033 | 0.2578 | 0.0733 | 0.1139 | 0.0281 | 0.052 | 0.0441 | 0.095 | 0.0828 | 0.039 |
|  | **Patients receiving invasive mechanical ventilation** | | | | | | | | | | | |
| AUROC (95% CI) | 0.71 (0.7-0.71) | 0.68 (0.63-0.74) | 0.68 (0.68-0.69) | 0.66 (0.6-0.72) | 0.75 (0.73-0.77) | 0.67 (0.56-0.78) | 0.66 (0.63-0.68) | 0.66 (0.62-0.69) | 0.66 (0.63-0.7) | NA | 0.58 (0.52-0.63) | 0.73 (0.69-0.77) |
| Calibration slope | 0.7885 | 0.6566 | 0.5943 | 0.5932 | 0.9195 | 0.6527 | 0.5544 | 0.505 | 0.5353 | NA | 0.3037 | 0.6671 |
| Calibration intercept | -1.556 | 1.845 | -0.0164 | 1.0062 | -1.4892 | 0.6066 | -0.577 | -0.8454 | -0.3087 | NA | -1.0506 | -0.0681 |
| O/E ratio (95% CI) | 0.36 (0.35-0.37) | 4.13 (3.9-4.37) | 1.11 (1.1-1.13) | 3.44 (3.17-3.73) | 0.36 (0.33-0.4) | 2.45 (2.03-2.95) | 0.96 (0.9-1.03) | 0.74 (0.66-0.83) | 1.2 (1.11-1.3) | NA | 1.38 (1.21-1.59) | 1.08 (0.99-1.18) |
| Brier score | 0.1448 | 0.4182 | 0.2204 | 0.3021 | 0.1408 | 0.2181 | 0.1535 | 0.1673 | 0.178 | NA | 0.127 | 0.1977 |

**Supplementary Table 9: Results from ICU mortality sensitivity analyses**

|  | **Aus & NZ** | **Bangladesh** | **Brazil** | **Ethiopia** | **Finland** | **Ghana** | **India** | **Malaysia** | **Nepal** | **Netherlands** | **South Africa** | **Uruguay** |  |
| --- | --- | --- | --- | --- | --- | --- | --- | --- | --- | --- | --- | --- | --- |
|  | **Complete case analysis** | | | | | | | | | | | | |
| AUROC (95% CI) | 0.78 (0.78-0.79) | 0.67 (0.63-0.71) | 0.81 (0.81-0.82) | 0.62 (0.56-0.69) | 0.81 (0.79-0.82) | 0.63 (0.48-0.79) | 0.67 (0.66-0.69) | 0.68 (0.64-0.71) | 0.66 (0.64-0.69) | 0.8 (0.79-0.8) | 0.63 (0.58-0.67) | 0.78 (0.75-0.8) |  |
| Calibration slope | 0.8761 | 0.5707 | 1.0494 | 0.4665 | 0.975 | 0.5791 | 0.6321 | 0.5575 | 0.5632 | 0.8564 | 0.4658 | 0.8488 |  |
| Calibration intercept | -1.3386 | 2.2997 | -0.8133 | 0.5309 | -1.3542 | 1.2024 | -0.9138 | -0.8657 | -0.6292 | -1.1228 | -0.7883 | -0.2162 |  |
| O/E ratio (95% CI) | 0.42 (0.4-0.43) | 6.37 (6.17-6.58) | 0.54 (0.53-0.55) | 2.97 (2.71-3.27) | 0.42 (0.38-0.45) | 3.36 (2.79-4.05) | 0.75 (0.71-0.79) | 0.73 (0.65-0.81) | 1.07 (1.01-1.14) | 0.52 (0.5-0.54) | 1.35 (1.19-1.54) | 0.96 (0.88-1.05) |  |
| Brier score | 0.109 | 0.53 | 0.0779 | 0.2781 | 0.1242 | 0.3468 | 0.0975 | 0.1505 | 0.1192 | 0.1384 | 0.1061 | 0.1409 |  |
|  | **Excluding all inter-hospital transfers** | | | | | | | | | | | | |
| AUROC (95% CI) | 0.82 (0.81-0.82) | 0.65 (0.62-0.68) | 0.82 (0.81-0.82) | 0.67 (0.61-0.72) | 0.81 (0.79-0.82) | 0.7 (0.6-0.79) | 0.7 (0.68-0.72) | 0.71 (0.68-0.74) | 0.68 (0.66-0.7) | 0.79 (0.79-0.8) | 0.64 (0.59-0.69) | 0.8 (0.78-0.83) |  |
| Calibration slope | 1.0172 | 0.4853 | 1.0465 | 0.5942 | 1.0496 | 0.7081 | 0.7102 | 0.6365 | 0.6181 | 0.9413 | 0.5026 | 0.9058 |  |
| Calibration intercept | -1.7081 | 1.6468 | -0.852 | 0.7701 | -1.6769 | 0.7559 | -0.9012 | -0.9407 | -0.6325 | -1.5576 | -0.7183 | -0.2873 |  |
| O/E ratio (95% CI) | 0.27 (0.26-0.28) | 6.77 (6.58-6.96) | 0.51 (0.5-0.52) | 3.14 (2.9-3.4) | 0.3 (0.27-0.33) | 2.54 (2.16-2.99) | 0.7 (0.66-0.74) | 0.66 (0.59-0.74) | 1.02 (0.96-1.08) | 0.35 (0.34-0.36) | 1.36 (1.18-1.56) | 0.89 (0.81-0.98) |  |
| Brier score | 0.0765 | 0.4523 | 0.0683 | 0.2528 | 0.1016 | 0.2144 | 0.0816 | 0.1263 | 0.1051 | 0.1174 | 0.1024 | 0.113 |  |

**Supplementary Figure 1: Participant flow diagram across all registries included in the primary analysis**

**Supplementary Figure 2: Participant flow diagram across all registries for the secondary (ICU mortality) analysis**
