# Supplementary material for: Evaluation of the SMS-ICU score for multicontinental use: A federated external validation study using critical care registry data
Source: Crit Care Resusc. 2026 Aug 13;28(3):100206. doi: 10.1016/j.ccrj.2026.100206 (PMC13499179; doi:10.1016/j.ccrj.2026.100206)
Supplement: Supplementary file 3 [file mmc3.docx]

**The authors and the ANZICS CORE management committee would like to thank clinicians, data collectors and researchers at the following contributing sites in Australia and New Zealand:**

Albury Base Hospital ICU, Alfred Hospital ICU, Alice Springs Hospital ICU, Angliss Hospital ICU, Armadale Health Service ICU, Ashford Community Hospital ICU, Auckland City Hospital CV ICU, Auckland City Hospital DCCM, Austin Hospital ICU, Ballarat Health Services ICU, Bankstown-Lidcombe Hospital ICU, Bathurst Base Hospital ICU, Bendigo Health Care Group ICU, Blacktown Hospital ICU, Bowral Hospital HDU, Box Hill Hospital ICU, Brisbane Private Hospital ICU, Broken Hill Base Hospital & Health Services ICU, Buderim Private Hospital ICU, Bunbury Regional Hospital ICU, Bundaberg Base Hospital ICU, Caboolture Hospital ICU, Cabrini Hospital ICU, Cairns Hospital ICU, Calvary Bruce Private Hospital HDU, Calvary Hospital (Canberra) ICU, Calvary Hospital (Lenah Valley) ICU, Calvary John James Hospital ICU, Calvary Mater Newcastle ICU, Calvary North Adelaide Hospital ICU, Calvary Wakefield Hospital (Adelaide) ICU, Campbelltown Hospital ICU, Canberra Hospital ICU, Casey Hospital ICU, Central Gippsland Health Service ICU, Christchurch Hospital ICU, Coffs Harbour Health Campus ICU, Concord Hospital (Sydney) ICU, Dandenong Hospital ICU, Dubbo Base Hospital ICU, Dunedin Hospital ICU, Echuca Regional Hospital HDU, Epworth Eastern Private Hospital ICU, Epworth Freemasons Hospital ICU, Epworth Geelong ICU, Epworth Hospital (Richmond) ICU, Fairfield Hospital ICU, Fiona Stanley Hospital ICU, Flinders Medical Centre ICU, Flinders Private Hospital ICU, Footscray Hospital ICU, Frankston Hospital ICU, Gold Coast Private Hospital ICU, Gold Coast University Hospital ICU, Gosford Hospital ICU, Gosford Private Hospital ICU, Goulburn Base Hospital ICU, Goulburn Valley Health ICU, Grafton Base Hospital ICU, Greenslopes Private Hospital ICU, Griffith Base Hospital ICU, Hawkes Bay Hospital ICU, Hervey Bay Hospital ICU, Hollywood Private Hospital ICU, Holmesglen Private Hospital ICU, Holy Spirit Northside Hospital ICU, Hornsby Ku-ring-gai Hospital ICU, Hurstville Private Hospital ICU, Hutt Hospital ICU, Ipswich Hospital ICU, John Fawkner Hospital ICU, John Flynn Private Hospital ICU, John Hunter Hospital ICU, Joondalup Health Campus ICU, Kareena Private Hospital ICU, Knox Private Hospital ICU, Latrobe Regional Hospital ICU, Launceston General Hospital ICU, Lingard Private Hospital ICU, Lismore Base Hospital ICU, Liverpool Hospital ICU, Logan Hospital ICU, Lyell McEwin Hospital ICU, Mackay Base Hospital ICU, Macquarie University Private Hospital ICU, Maitland Hospital HDU/CCU, Maitland Private Hospital, Manly Hospital & Community Health ICU, Manning Rural Referral Hospital ICU, Maroondah Hospital ICU, Mater Adults Hospital (Brisbane) ICU, Mater Health Services North Queensland ICU, Mater Private Hospital (Brisbane) ICU, Mater Private Hospital (Sydney) ICU, Melbourne Private Hospital ICU, Middlemore Hospital ICU, Mildura Base Hospital ICU, Monash Medical Centre-Clayton Campus ICU, Mount Hospital ICU, Mount Isa Hospital ICU, National Capital Private Hospital ICU, Nelson Hospital ICU, Nepean Hospital ICU, Nepean Private Hospital ICU, Newcastle Private Hospital ICU, Noosa Hospital ICU, North Shore Hospital ICU, North Shore Private Hospital ICU, North West Private Hospital ICU, North West Regional Hospital (Burnie) ICU, Northeast Health Wangaratta ICU, Northern Beaches Hospital, Norwest Private Hospital ICU, Orange Base Hospital ICU, Peninsula Private Hospital ICU, Pindara Private Hospital ICU, Port Macquarie Base Hospital ICU, Prince of Wales Hospital (Sydney) ICU, Prince of Wales Private Hospital (Sydney) ICU, Princess Alexandra Hospital ICU, Queen Elizabeth II Jubilee Hospital ICU, Redcliffe Hospital ICU, Robina Hospital ICU, Rockhampton Hospital ICU, Rockingham General Hospital ICU, Rotorua Hospital ICU, Royal Adelaide Hospital ICU, Royal Brisbane and Women's Hospital ICU, Royal Darwin Hospital ICU, Royal Hobart Hospital ICU, Royal Melbourne Hospital ICU, Royal North Shore Hospital ICU, Royal Perth Hospital ICU, Royal Prince Alfred Hospital ICU, Ryde Hospital & Community Health Services ICU, Shoalhaven Hospital ICU, Sir Charles Gairdner Hospital ICU, South East Regional Hospital ICU, South West Healthcare (Warrnambool) ICU, Southern Cross Hospital (Wellington) ICU, Southland Hospital ICU, St Andrew's Hospital (Adelaide) ICU, St Andrew's Hospital Toowoomba ICU, St Andrew's Private Hospital (Ipswich) ICU, St Andrew's War Memorial Hospital ICU, St George Hospital (Sydney) ICU, St George Private Hospital (Sydney) ICU, St John of God (Berwick) ICU, St John Of God Health Care (Subiaco) ICU, St John Of God Hospital (Ballarat) ICU, St John of God Hospital (Bendigo) ICU, St John Of God Hospital (Geelong) ICU, St John Of God Hospital (Murdoch) ICU, St John of God Midland Public & Private ICU, St Vincent's Hospital (Melbourne) ICU, St Vincent's Hospital (Sydney) ICU, St Vincent's Hospital (Toowoomba) ICU, St Vincent's Private Hospital (Sydney) ICU, St Vincent's Private Hospital Fitzroy ICU, Sunnybank Hospital ICU, Sunshine Coast University Hospital ICU, Sunshine Coast University Private Hospital ICU, Sunshine Hospital ICU, Sutherland Hospital & Community Health Services ICU, Sydney Adventist Hospital ICU, Sydney Southwest Private Hospital ICU, Tamworth Base Hospital ICU, Taranaki Health ICU, Tauranga Hospital ICU, The Bays Hospital ICU, The Chris O’Brien Lifehouse ICU, The Memorial Hospital (Adelaide) ICU, The Northern Hospital ICU, The Prince Charles Hospital ICU, The Queen Elizabeth (Adelaide) ICU, The Townsville Hospital ICU, The Valley Private Hospital ICU, The Wesley Hospital ICU, Timaru Hospital ICU, Toowoomba Hospital ICU, Tweed Heads District Hospital ICU, University Hospital Geelong ICU, Victorian Heart Hospital ICU, Wagga Wagga Base Hospital & District Health ICU, Waikato Hospital ICU, Wairau Hospital ICU, Warringal Private Hospital ICU, Wellington Hospital ICU, Werribee Mercy Hospital ICU, Western District Health Service (Hamilton) ICU, Western Hospital (SA) ICU, Western Private Hospital ICU, Westmead Hospital ICU, Westmead Private Hospital ICU, Whakatane Hospital ICU, Whangarei Area Hospital, Northland Health Ltd ICU, Wimmera Health Care Group (Horsham) ICU, Wollongong Hospital ICU, Wollongong Private Hospital ICU, Women's and Children's Hospital PICU, Wyong Hospital ICU

**The authors and BACIR committee would like to thank clinicians, data collectors and researchers at the following Bangladeshi site: Chittagong Medical College. The authors would also like to thank Professor Aniruddha Ghose FCPS, MD, FRCP.**

**The authors would like thank all who contributed to ORCHESTRA-Brazil.**

**Steering Committee:** Marcio Soares, Fernando Augusto Bozza; Jorge Ibrain Figueira Salluh; Pedro Martins Pereira Kurtz, Gastón Burghi, Fernando G. Zampieri

**Research Coordinators:** Aline Reis da Silva Antunes, Grazielle Viana Ramos

**Statisticians:** Leonardo dos Santos Lourenco Bastos, Lunna Perdigão Borges, Thaís Machado, Gabriel Miranda

**Centers and Investigators**

**Brazil: Alagoas -** *Hospital Memorial Arthur Ramos*: Maria Valéria de Carvalho Wanderley; Aline dos Santos Carvalho, Amanda Ribeiro de Mendonça Picone, Morghana Aparecida Rodrigues Ferreira, Bruna Xavier Brito; **Amazonas -** *Hospital Delphina Rinaldi Abdel Aziz*: Liane Cavalcante, Irina Jerez Jerez, Yudermys Amezaga Santana, Helena Alvarenga Sardenberg, Edna Freitas Martins, Marcia Lidiane Vasconcelos Dias Amorim; **Bahia -** *Hospital Cardiopulmonar*: Edson Silva Marques Filho; Antônio Fernando Borba Fróes Júnior, Daniel Beckerath da Silva Leitão; *Hospital Geral Cleriston Andrade:* Lúcio Couto de Oliveira Junior, Patrick Harrison Santana Sampaio, Renata Nunes de Oliveira; Diego Venicio Santos Argolo, Vanessa Freitas Vital, Bruno Cunha de Oliveira, João Victor Brito do Vale, Ramaiana de Jesus Gonzaga Cavalcante, Lúcio Couto de Oliveira Junior, Joaquim Agatângelo de Souza, Alberto Manoel Sarkis de Oliveira, Larissa Fernandes Oliveira, Daniela Cunha de Oliveira, Ricardo Peixoto Oliveira, Paulo Henrique Panelli Ferreira, Jamyllo Sales Brito, Elissama de Jesus Sena Reis, Vinicius Silva Oliveira, Geiza Santana Vidal; *Hospital da Cidade:* André Luiz Nunes Gobatto; Sydney Agareno de Souza Filho, Luciana Sampaio de Mattos Palmeira, Licurgo Pamplona Neto, Livia Magalhães Costa Castro; *Hospital Unimed Baia de Todos os Santos*: Lúcio Couto de Oliveira Junior; Joaquim Paulo Castro de Santana, Diego Venício Argolo, Patrick Harrison Sampaio, Joaquim Agatângelo Sousa, Tarsila Correia Ribeiro; **Ceará -** *Hospital Monte Klinikum*: David Theophilo Araújo; Victor Souza Cravo, Douglas Holanda Campos Filho, Ricardo Eustáquio Magalhães, Manuella Meireles Victor Souza Cravo, Pereira Gadelha Santos, Francisca Jane Gomes de Oliveira; **Distrito Federal**: *Hospital Anchieta:* Marcelo Oliveira Maia; Adriano Drummond, Noara Barros, Carla Moggia, Ivna Asfor; *Hospital Santa Luzia:* Marcelo Oliveira Maia; Fábio Amorim, Carlos Darwin; *Hospital DF Star*: Antônio Aurélio Fagundes Jr; *Hospital Brasília Unidade Águas Claras*: Vinicius Machado Santos; Pedro Henrique Rosa da Silveira, Tiago Samuel Lima Pontes, Flavio Carvalho dos Santos; **Espírito Santo -** *Hospital Meridional:* Marcus Vinicius A. Leitão; Lucas Resende Aniceto, Lucas Dornelas F. Machado Silva, Shayra Pansini Souza, Frederico Machado de Siqueira; *Hospital Unimed Vitória*: Eliana Bernardete Caser; **Goiás -** *Hospital Municipal de Aparecida de Goiânia – HMAP*: Joan Rodrigues de Castro; Maurício Mascarenhas Boaventura, Cristian Andrade Garcia, Renata de Souza Cyrino, Gean Carlos Alves Moraes; *Hospital Israelita Albert Einstein – Goiânia*: Ângelo Antônio Gomes de Carvalho; *Centro Estadual de Reabilitação e Readaptação Dr. Henrique Santillo – CRER*: Eduardo Vilela, Ciro Bruno Silveira Costa, Priscila Martins Pereira, Ludmila Gomes dos Santos, Ronycley Rocha Rezende, Fabianne Silveira Cardoso; *HUGOL – Hospital Estadual de Urgências Governador Otávio Lage de Siqueira*: Antônio Elias Lopes, Flávio Augusto Castro, Gustavo Prudente, Alex Linhares, Gabriel Fogaça, Igor Ferreira Capelleti; **Maranhão** - *Hospital São Domingos:* José Raimundo Azevedo; Luis Eduardo França Tupinambá Junior; *UDI Hospital*: Alexandre Guilherme Ribeiro de Carvalho; Edilene Coelho de Souza Novaes, Lucas Akira Costa Hirai, Daniel Wagner de Castro Lima Santos, Louise Aline Romão Gondim, Tânia Karla Sousa Nogueira Rosa; *Hospital Maranhense:* Filipe Sousa Amado; *Hospital do Cancer do Maranhão Tarquinio Lopes Filho*: Gustavo Teixeira Alves; Sara Vieira Nascimento; *Hospital de Traumatologia e Ortopedia do Maranhão*: Luciana Sousa Silva; Rosinete Andrade Ferreira; **Minas Gerais** - *Hospital Metropolitano Doutor Célio de Castro*: Luidy Luciano Cardoso; Roberto Sydney, Paolo Tótola, Bruno Resende, Diogo Madeira; *Hospital Felício Rocho*: Rogério de Castro Pereira; Thais de Paula Guimarães, Sinval Lins Silva, Daniel Fontes, Débora Avelar Afonso da Silva, Ana Clara Ferreira Amâncio Pereira; *Hospital das Clínicas da UFMG*: Saulo Fernandes Saturnino; *Pró-Saúde Hospital Metropolitano Vale do Aço:* Luiz Henrique de Araújo Pereira Costa; Norberto de Sá Neto, Rodrigo Silveira Machado, José Roberto Batista; *Hospital Risoleta Tolentino Neves*: Marco Aurélio Reis, Pulchéria Leôncio Pereira Araujo, Camila Martins Ramos; **Pará -** *Hospital Adventista de Belém*: Edgar de Brito Sobrinho; **Paraíba -** *Hospital Nossa Senhora das Neves*: Paulo César Gottardo; Elbia Assis Wanderley, Katyucia Egito de Araújo Urquisa, Andreia Cristina Fumagalli Cainelli, Vinilton Leandro Ferreira; *Hospital Universitário Lauro Wanderley - UFPB/EBSERH*: Ciro Leite Mendes; Igor Mendonça do Nascimento; **Paraná -** *Hospital Santa Cruz*: Hipolito Carraro Jr. *Hospital São Marcelino Champagnat*: Viviane Bernardes de Oliveira Chaiben; Maria Lygia Minney, Andressa de Souza Bertoldi, Karen Fernandes de Moura, Gustavo Henrique dos Santos Silva, Bruno Alcantara Gabardo; *Hospital Municipal Padre Germano Lauck*: Roberto de Almeida; Guilherme Ribeiro, Mirian Liliana Insfrán Franco; *Hospital Universitário Cajuru*: Viviane Bernardes de Oliveira Chaiben; Giovanna Cerri Lessa, Victor Hugo Santana Lourenço de Lima, Leandro Bressianini Jurkonis, Marcos vinicius Streit, Gabriela Martins Teixeira; *Hospital Nossa Senhora das Graças:* Iara Buselato Chen; Vanessa Padilha Tomba, Marta Ângela Brandão; *Hospital Araucária de Londrina*: Cintia Magalhães Carvalho Grion; *Hospital Universitário Regional do Norte do Paraná*: Cintia Magalhães Carvalho Grion; **Pernambuco -** *Real Hospital Português*: Mário Diego Teles Correia; *Hospital Santa Joana Recife*; Gustavo Trindade Henriques Filho; Arthur Henrique Ribeiro do Valle de Faria, Marcos Antonio Cavalcanti Gallindo, Carlos Eduardo Ferraz Freitas, Ana Flávia de Melo Campos, Danielle Ferraz de Oliveira Aguiar; **Rio de Janeiro -** *Hospital Copa D'Or:* Aloysio Saulo Beiler; *Hospital Quinta D'Or*: Alexandre Coscia, Laura Herranz Prinz, Juliana Gurgel da Silveira, Cristiane Belo, Soraya Pulier; Joao Vitor Bessa, Juliana Gravina, Thiago Prata, Joyce Roma Lucas de Silva, Alessandra Longo, Lorena fonseca, Alexandra Gonçalves, Marcelo Cruzick; *Hospital Estadual Alberto Torres*: Ulisses de Oliveira Melo; *Hospital Nossa Senhora do Carmo*: Paula Figueiredo Natel; Raphaela Mannarino, Nelson Poubel, Amir Gonçalves Neto, Marcelo Motta; *Complexo Américas*: Victor Cravo; Emmanuel Salgueiro; *Hospital Norte D'Or*: Douglas Quintanilha Braga; Sergio Teixeira Sant'Anna Júnior, Gustavo Caniné da Costa, Renata Ribeiro Leite do Amaral, Camila Lima Ferreira da Costa, Thalita Montenegro Prieto Lloret; *Hospital Barra D´Or*: Walter Homena Jr, Marcelo de Sousa Santino, Gloria Adriana Rocha Martins; José Alexandre Espósito Panaro, Francisco Gonçalves Gabriel, Paula Gorgulho, Marcelo Felix, Luciana Freitas de Oliveira; *Hospital Pasteur*: Pedro Henrique Barbosa D' Almeida; Carlos Eduardo Brandão, Raquel Pereira De Farias Evangelista, Danessa Moreira Rodrigues; *Hospital Rios D'Or*: Alessandra Alves; *Hospital Caxias D'Or*: Eric Perecmanis; *Hospital Niterói D'Or*: Ricardo Turon	Bruno Guimarães; *Hospital Oeste D'Or*:Guilherme Brenande Alves Faria, Márcia Adélia de Magalhães Menezes; Liliane Rodrigues de Mendonça, Reinaldo Campos Rodrigues, Caros Henrique Ferreira Ramos, Rosa Imaculada Stancato, Joyce Andrade; *Hospital São Lucas Copacabana*: Aline Affonso, Laura Herranz Prinz; Bruno Gonçalves; *Hospital Municipal Evandro Freire*: Fabio Basilio Fernandes dos Santos; *Hospital Adventista Silvestre*: Fernando Santiago Montenegro; *Hospital Badim*: Edmundo de Oliveira Tommasi; Alexandra Goncalves da Silva, Alexandre Vaz Scotti, Jefferson dos Santos Daros, Fábio Guilherme Santoro; *Hospital Samer:* Henrique Miller Balieiro; Marcelo Namen; *Hospital Copa Star:* Pedro Kurtz, Rodolfo Espinoza; Rafaella Pottes; *Hospital de Clínicas de Jacarepaguá*: Fernando Alves Rocha; Simone Cristina Santos De Lira, Ana Paula Figueiredo De Carvalho, Carlos Eduardo Brandão; *Hospital Gloria D'Or*: Cecília Magno; Nathane Santanna; *Hospital Municipal da Japuiba*: Viviane Bogado Leite Torres; Michelle Cristina Ferreira Soares, Beatriz Victoria Correia Ferreira, Josiane Cristina da Silva, Julia Monteiro Novaes, Tatiany Lopes Lessa; *Hospital de Clínicas Mario Lioni*: Fernando Santana Pinto; Carlos Eduardo Brandão, Juliana Sales De Ornelas; *Hospital e Maternidade Santa Lúcia:* Eduardo Costa Pinto; Carlos Eduardo Brandão, Elen Silva Ferreira, Marcelo Alonso De Barros Correia; *Hospital da Fundação Eletronuclear de Assistência Médica – FEAM*: Viviane Bogado Leite Torres, Mariana Rodrigues Farias Andrade; *Instituto Nacional de Câncer - Hospital do Câncer II:* Rodolfo Espinoza, Rafael Mandarino; **Rio Grande do Sul -** *Hospital Mãe de Deus*: Lucas Vieira de Souza; *Hospital de Clínicas de Porto Alegre:* Fabiano Márcio Nagel; Márcio Manozzo Boniatti; *Hospital Dom João Becker*: Michael Milman; **Santa Catarina -** *Hospital Municipal Ruth Cardoso*: Pedro Salomão Dias, Cesar Augusto Meirelles de Almeida, Eduardo Bellotto, Pablo Wanglon Richter; **São Paulo –** *Hospital A.C. Camargo*: Antônio Paulo Nassar Jr; Silvana Soares dos Santos; *Hospital e Maternidade Brasil:* Fabio de Carvalho Mauricio; Tatiana Gozzi Pancev Toledo, Fernando Ramos Pellegrini, Manoela Prado Pasqualucci Esposito; *Hospital Municipal Moysés Deutsch*: Niklas Soderberg Campos; Bruna Achar Soderberg Campos, Paula Geraldes David João, Luiz Adriano Esteves, Petrus Söderberg Campos, Luciana Silveira de Oliveira; *Hospital Nove de Julho*: Marcos Soares Tavares; Celso Madeira Padovesi, Antônio Paulo Martins Ramos Filho; *IAMSPE - Hospital do Servidor Público Estadual*: Ederlon Alves de Carvalho Rezende; Ellen Pierre Oliveira, Caio Gouvêa Jaoude, Vânia Quinato Malacize, Mateus Demarchi Gonsalves; *Hospital de Base*: Suzana Ajeje Margareth Lobo; Luana Fernandes Machado, Juliana Devós Syrio Martinez, Marcio Mussolino de Queiroz, Neymar Elias de Oliveira, Silvia Prado Minhoto Teixeira Ramin, Vanessa Aparecida Maziero Santana; *Hospital São Luiz Morumbi*: José Célio Vieira Brandão; Adriana Peixoto Gelmetti de Barros; *Hospital ViValle*: Flavio Rodrigues de Sousa; Felipe de Jesus Gonçalves; *Hospital São Luiz Itaim*: Mariza Silva Ramos Loesch, Thiago Gomes Romano; *Hospital Villa-Lobos*: Carlos Antônio Carvalho Ribeiro; Flávio Geraldo Rezende de Freitas; *Hospital Israelita Albert Einstein – Morumbi*: Thiago Domingos Corrêa; *Hospital do Coração HCOr*: Edson Renato Romano; Rosianne Vasconcelos; *Hospital Assunção*: Silvia Regina Ramos; *Hospital Metropolitano*: Eduardo Augusto Pessoa Gomes; Carlos Eduardo Brandão, Liliane Alves Feitoza Turci, Aline Nunes Lobo Bueno; *Hospital da Luz*: Daniel Almeida Schettini; Carlos Eduardo Brandão, Vaneska Mazzini, Lilian Louise Coelho Pereira; *Hospital Sírio Libanês - Bela vista SP:* Laerte Pastore Jr.; Fernando José da Silva Ramos, Naira Lima Matos, Clara Esther Maciel dos Santos, Bruno Martins Tomazini, Nilda Rosa de Oliveira Prado; *Hospital Panamericano*: Eduardo Costa Pinto; Carlos Eduardo Brandão, Vanessa Ribeiro Pardauil, Cristiane Cunha Da Silva Rosa; *Hospital Vitória Anália Franco*: Guilherme Rossini; Carlos Eduardo Brandão, Mariana Celeghini Santiago Gosik, Priscilla Belchior Gonzalez; *Serviço Social da Ind. do Papel Papelão e Cortiça Do Est. São Paulo - Hospital e Maternidade SEPACO:* Nathaly Fonseca Nunes; Daniela Boschetti, Flávio Geraldo Rezende de Freitas; *Hospital Alvorada Moema*: Amanda Mota de O. Veiga; Carlos Eduardo Brandão; *Hospital Carlos Chagas*: Fernando Jose Bricks; Carlos Eduardo Brandão, Caroline Scodelario Cortes, Francisco Afranio Miranda; *Hospital São Paulo*: Flavia Ribeiro Machado; *Hospital Santa Helena*: Alessandra Borges Mendes Gonzaga; Carlos Eduardo Brandão, Tatiane Oliveira Luiz, Liliane Lemos; *Hospital Japonês Santa Cruz:* Thiago Miranda Lopes de Almeida; Sérgio Sônego Fernandes, Carlos Antônio Carvalho Ribeiro, Flávio Geraldo Rezende de Freitas; *Hospital Samaritano Higienópolis*: Marcos Cairo Vilela; Barbara Cristina de Abreu Pereira, Fernando Antonio Alvares da Costa, Leonardo Fernando Ferrari Nogueira , Natalia Lopes Ferreira, Luciana Rosa Fidelis; *BP – A Beneficência Portuguesa de São Paulo – Unidade Mirante*: Viviane Cordeiro Veiga; *Hospital Ana Costa*: Fernanda Rodrigues Martins Masteguim; Carlos Eduardo Brandão, Thiago Santos da Silva, Fatima Cristina Andrade Rodrigues; *Hospital Paulistano*: Alder Costa Garcia da Silveira; Carlos Eduardo Brandão, Debora Prudencio E Silva, Helenice De Paula Vieira; *Santa Casa de Piracicaba*: Rafael Angelo Tineli; Luciana Marcolino Tineli; *Hospital Beneficência Portuguesa de Ribeirão Preto*: Marcus Antônio Ferez; Franceliana Prado Barbosa Sgobi, Fernanda Valéria Ramos Paiolo; *Hospital e Maternidade Ribeirão Pires*: Fabio de Carvalho Mauricio, Tatiana Gozzi Pancev Toledo, Fernando Ramos Pellegrini, Manoela Prado Pasqualucci Esposito; *Hospital dos Fornecedores de Cana de Piracicaba*: Renata Lopes Basso; Rafael Angelo Tineli; *Hospital Pitangueiras*: Márcio Shimabuku e Silva; Carlos Eduardo Brandão, Felipe Neves Marcelino, Alex Oliveira; *Hospital Vila Nova Star:* Thiago Gomes Romano; *Hospital São Bernardo:* Ana Paula Mascarelli Amaral; Carlos Eduardo Brandão, Maria De Fatima Silva De Almeida, Janaina De Cassia Lopes Dos Santos; *Hospital Novo Atibaia*: Rubens Sergio da Silva Franco; Amauri Francisco De Marchi Bemfica, Walter Carlos Girardelli Baptista, Manoela Moreira de Sousa, Aline Ribeiro Moreira, Juliana Regina Berto Wada; *Hospital de Clinicas de Caieiras*: Volnei Martins Castanho; Carlos Eduardo Brandão, Priscila Gonzaga dos Santos, Elaine Santana da Silva, Marciely Alves Ramalho de Oliveira, Viviane Gimenez Rossini , Imara Jacinto de Azevedo Rios; *Hospital Ipiranga*: Rafael Di Domenico Mattos; Carlos Eduardo Brandão, Fabricio Campos Morais Moreira, Julienne Garcia Rissatti, Luiz Henrique Costa Garcia; *Hospital Santo Amaro:* Sanmya Danielle Rodrigues dos Santos; Carlos Eduardo Brandão, Dominique Almeida Cruz, Bárbara Fialdini Von Ah; *Hospital e Maternidade Ipiranga Arujá*: Rafael Di Domenico Mattos, Carlos Eduardo Brandão, Gisele Aparecida Cardoso; *Hospital das Clínicas da Faculdade de Medicina da Universidade de São Paulo*: Leandro Utino Taniguchi; Bruno Adler Maccagnan Pinheiro Besen; Roberta Muriel Longo Roepke; Pedro Fortes Osório Bustamante; Ana Clara Marcondes Dobre; *Unimed Capivari*: Rafael Angelo Tineli; Daniela Mazzini Quagliato Azanha; *Hospital IFOR*: Sílvia Regina Ramos.

**The authors would like to thank the staff and data collectors at the following sites in Ethiopia:**

- AaBET Hospital GICU
- St Paul's Hospital GICU

**The authors are grateful to the clinical staff and data integrity specialists at the following hospitals that contribute data to the Finnish Intensive Care Consortium registry:**

Helsinki University Central Hospital, Espoo and Helsinki

Kuopio University Hospital, Kuopio

Oulu University Hospital, Oulu

Tampere University Hospital, Tampere

Turku University Central Hospital, Turku

Central Finland Central Hospital, Jyväskylä

Kainuu Central Hospital, Kajaani

Kanta-Häme Central Hospital, Hämeenlinna

Keski-Pohjanmaa Central Hospital, Kokkola

Kymenlaakso Central Hospital, Kotka

Lappi Central Hospital, Rovaniemi

Länsi-Pohja Central Hospital, Kemi

Mikkeli Central Hospital, Mikkeli

North Karelia Central Hospital, Joensuu

Päijät-Häme Central Hospital, Lahti

Satakunta Central Hospital, Pori

Seinäjoki Central Hospital, Seinäjoki

South Karelia Central Hospital, Lappeenranta

Vaasa Central Hospital, Vaasa

**The authors would like to thank the staff and data collectors at the Ghana critical care registry.**

**We would like to thank all the sites, site leads, and research teams that are part of the Indian Registry of IntenSive care (IRIS). We would like to acknowledge the following sites who contributed to this work:**

| AIIMS, Bhubaneswar, Odisha | Swagata Tripathy |
| --- | --- |
| AIIMS, Bhubaneswar, Odisha | Kasturi Sanyal |
| AIIMS, Bhubaneswar, Odisha | Sunil Kumar Nayak |
| Amrita Institute of Medical Sciences, Kochi, Kerala | Zubair Umer Mohamed |
| Amrita Institute of Medical Sciences, Kochi, Kerala | Anna Paul |
| Apollo Cancer Institute, Chennai | Dedeepiya Devaprasad |
| Apollo Cancer Institute, Chennai | Rohit Aravindakshan Kooloth |
| Apollo Cancer Institute, Chennai | Sandeep Shyamsundar |
| Apollo Cancer Institute, Chennai | Vijay Vasu |
| Apollo Cancer Institute, Chennai | Deepak Raj Sampath Kumar |
| Apollo Cancer Institute, Chennai | Sowmiya Panneer Selvam |
| Apollo First Med Hospital, Chennai | Meghena Mathew |
| Apollo First Med Hospital, Chennai | Preethika Ravichandran |
| Apollo First Med Hospital, Chennai | Lakshmi Narashimhan Srinivasan |
| Apollo Main Hospital, Chennai | Bharath Kumar Tirupakuzhi Vijayaraghavan |
| Apollo Main Hospital, Chennai | Ramakrishnan Nagarajan |
| Apollo Main Hospital, Chennai | Augustian James |
| Apollo Main Hospital, Chennai | Sruthi Mano |
| Apollo Main Hospital, Chennai | Mithilesh Devaraj |
| Apollo Proton Cancer Centre, Chennai | Raymond Dominic Savio |
| Apollo Proton Cancer Centre, Chennai | Suresh Kumar Sundaramurthy |
| Apollo Proton Cancer Centre, Chennai | Vinisha Varadharajan |
| Apollo Speciality Hospital – OMR, Chennai | Pratheema Ramachandran |
| Apollo Speciality Hospital – OMR, Chennai | Swedha Vasudevan |
| Apollo Speciality Hospital – OMR, Chennai | Priyadharshini Vasudevan |
| Apollo Speciality Hospital, Vanagaram, Chennai | Ebenezer Rabindrarajan |
| Apollo Speciality Hospital, Vanagaram, Chennai | Vanishree Chandran |
| Apollo Speciality Hospital, Vanagaram, Chennai | Mohana Priya Sachidhanandam |
| Dr. Kamakshi Memorial Hospital, Pallikaranai, Chennai | Devachandran Jayakumar |
| Dr. Kamakshi Memorial Hospital, Pallikaranai, Chennai | Dinesh Krishnamoorthy |
| Dr. Kamakshi Memorial Hospital, Pallikaranai, Chennai | Vignesh Rajasekaran |
| Dr. Kamakshi Memorial Hospital, Pallikaranai, Chennai | Ramayana Manohar Avi |
| Dr. Kamakshi Memorial Hospital, Pallikaranai, Chennai | Lavanya Elumalai |
| Dr. Kamakshi Memorial Hospital, Pallikaranai, Chennai | Sucharita Yanamandra |
| ISPAT General Hospital, Rourkela, Odisha | Chinni Krishna Kasi |
| ISPAT General Hospital, Rourkela, Odisha | Durga Madhab Tripathi |
| Pushpagiri Medical College Hospital, Tiruvalla, Kerala | Mathew Pulicken |
| Tata Memorial Hospital, Mumbai | Atul P Kulkarni |
| Tata Memorial Hospital, Mumbai | Sheila Nainan Myatra |
| Tata Memorial Hospital, Mumbai | Anjana Shrivastava |
| Tata Memorial Hospital, Mumbai | Nikita Kulaye |
| Tata Memorial Hospital, Mumbai | Karuna Panahle |
| Vijaya Medical and Educational Trust, Chennai | Balaji Venkatachalam |
| Vijaya Medical and Educational Trust, Chennai | Pavan kumar Vecham |
| Vijaya Medical and Educational Trust, Chennai | Mariswari Raja |

**The authors would like to thank the staff and data collectors at the following sites in Malaysia:**

IIUMMC @ SASMEC GICU

University Malaya Medical Centre GICU

Hospital Universiti Sains Malaysia Mixed ICU

Hospital Universiti Sains Malaysia SICU

**The authors would like to thank the staff and data collectors at the following sites in Nepal:**

Tribhuvan University Teaching Hospital Covid ICU (ICU B)

B & C Hospital GICU

Chitwan Medical College MICU

Nepal Mediciti Hospital MDICU

Tribhuvan University Teaching Hospital MICU

Om Hospital GICU

Tribhuvan University Teaching Hospital GICU( ICU-A)

Hospital for Advanced Medicine and Surgery (HAMS) Closed ICU

Grande International Hospital Closed mixed ICU

B & B Hospital GICU

Karuna Hospital GICU

Nidan Hospital GICU

**The authors and the Stichting NICE would like to express their sincere gratitude to all clinicians and data collectors from participating intensive care units throughout the Netherlands:**

Admiraal De Ruyter Ziekenhuis	Maasziekenhuis Pantein
Albert Schweitzer Ziekenhuis	Martini Ziekenhuis
Alrijne Zorggroep	Meander Medisch Centrum
Amphia Ziekenhuis	Medisch Spectrum Twente
Amstelland	Máxima MC
Amsterdam UMC	Nij Smellinghe
Antoni van Leeuwenhoek	Noordwest Ziekenhuisgroep

Antonius Zorggroep	Onze Lieve Vrouwe Gasthuis (OLVG)
Beatrixziekenhuis Rivas Zorggroep	Ommelander Ziekenhuis Groningen
Bernhoven	Radboud UMC
BovenIJ Ziekenhuis	Reinier de Graaf Gasthuis
Bravis Ziekenhuis	Rijnstate ziekenhuis
Canisius Wilhelmina Ziekenhuis	Rivierenland
Catharina Ziekenhuis	Rode Kruis Ziekenhuis
Deventer Ziekenhuis	Saxenburgh Groep
Diakonessenhuis	Slingeland Ziekenhuis
Dijklander ziekenhuis	Spaarne Gasthuis
Elisabeth Ziekenhuis - TweeSteden	St. Anna Ziekenhuis
Elkerliek Ziekenhuis	St. Antonius Ziekenhuis
Erasmus Medisch Centrum	St. Jans-Gasthuis
Flevoziekenhuis	St. Jansdal
Franciscus Ziekenhuis	Streekziekenhuis Koningin Beatrix
Frisius MC	Tergooiziekenhuizen
Gelderse Vallei	Treant Zorggroep
Gelre Ziekenhuis	UMC Groningen
Haaglanden MC	UMC Leiden
HagaZiekenhuis	UMC Maastricht
Het Groene Hart Ziekenhuis	UMC Utrecht
IJsselland Ziekenhuis	Van Weel Bethesda Ziekenhuis
Ikazia Ziekenhuis	VieCuri Medisch Centrum
Isala	Wilhelmina Ziekenhuis
Jeroen Bosch Ziekenhuis	Zaans Medisch Centrum
LangeLand Ziekenhuis	Ziekenhuisgroep Twente
Laurentius Ziekenhuis	ZorgSaam Zeeuws-Vlaanderen
Maasstad Ziekenhuis	Zuyderland


**The authors would like to thank the ICU teams and the data collectors, Yumna Beukes, Esihle Gili and Kieyaam Johnson, at Groote Schuur Hospital, Cape Town.**

**The authors and the Uruguayan management committee would like to thank clinicians, data collectors, and researchers at the contributing sites in Uruguay, with the support of the Sociedad Uruguaya de Medicina Intensiva (SUMI):**

UCI del Hospital Maciel, UCI del Hospital de Clínicas, UCI del Sanatorio Americano, UCI del Hospital Evangélico, UCI del Hospital Policial, UCI del Hospital Pasteur, UCI de COMECA, UCI de CRAMI, UCI de CAAMEPA, UCI del Sanatorio Mautone, UCI del Sanatorio Cantegril, UCI del Hopistal de Maldonado, UCI de COMERO, UCI del Hospital de Rocha, UCI del Hospital de Melo, UCI de CAMEDUR, UCI de AMSJ, UCI de CAMEC, UCI de CAMOC, UCI del Hospital de Mercedes, UCI del Hospital de Paysandú, UCI de SMQS, UCI de GREMEDA.
